# A phase II clinical trial of toripalimab in advanced solid tumors with polymerase epsilon/polymerase delta (*POLE/POLD1*) mutation

**DOI:** 10.1038/s41392-024-01939-5

**Published:** 2024-09-02

**Authors:** Ying Jin, Run-Jie Huang, Wen-Long Guan, Zhi-Qiang Wang, Zong-Jiong Mai, Yu-Hong Li, Jian Xiao, Xing Zhang, Qi Zhao, Shi-Fu Chen, Ming Liu, Yan-Xia Shi, Feng Wang, Rui-Hua Xu

**Affiliations:** 1 Department of Medical Oncology, Sun Yat-sen University Cancer Center, State Key Laboratory of Oncology in South China, Guangdong Provincial Clinical Research Center for Cancer, Sun Yat-sen University, Guangzhou, 510060 People’s Republic of China; 2 Research Unit of Precision Diagnosis and Treatment for Gastrointestinal Cancer, Chinese Academy of Medical Sciences, Guangzhou, 510060 People’s Republic of China; 3 Department of Oncology, The Fifth Affiliated Hospital of Sun Yat-sen University, Zhuhai, 519000 People’s Republic of China; 4 Department of Medical Oncology, Guangdong Provincial People’s Hospital (Guangdong Academy of Medical Sciences), Southern Medical University, Guangzhou, 510100 People’s Republic of China; 5 Department of Medical Melanoma and Sarcoma, Sun Yat-sen University Cancer Center, State Key Laboratory of Oncology in South China, Collaborative Innovation Center for Cancer Medicine, Sun Yat-sen University, Guangzhou, 510060 People’s Republic of China; 6 Bioinformatic Platform, Sun Yat-sen University Cancer Center, State Key Laboratory of Oncology in South China, Collaborative Innovation Center for Cancer Medicine, Sun Yat-sen University, Guangzhou, 510060 People’s Republic of China; 7 HaploX Biotechnology, Shenzhen, 518000 People’s Republic of China

**Keywords:** Cancer therapy, Predictive markers

## Abstract

Patients carrying mutations in polymerase epsilon/polymerase delta have shown positive responses to immune checkpoint inhibitors. Yet, prospective trials exploring the efficacy in those with polymerase epsilon/polymerase delta mutations are still lacking. A phase II clinical trial was initiated to evaluate the efficacy of toripalimab, a humanized IgG4K monoclonal antibody to human PD-1, in patients with advanced solid tumors with unselected polymerase epsilon/polymerase delta mutations but without microsatellite instability-high. A total of 15 patients were enrolled, 14 of whom were assessed for treatment efficacy. There was a 21.4% overall response rate, with a disease control rate of 57.1%. The median overall survival and median progression-free survival were 17.9 (95% CI 13.5-not reach) months and 2.5 (95% CI 1.4-not reach) months, respectively. For patients with exonuclease domain mutations, the objective response rate was 66.7% (2/3), with a disease control rate of 66.7% (2/3). For those with non-exonuclease domain mutations, the rates were 9.1% (1/11) and 54.5% (6/11), respectively. Notably, patients with *PBRM1* gene mutations exhibited a high response rate to toripalimab at 75.0% (3/4). This study showed that neither the exonuclease domain mutations nor non-exonuclease domain mutations could fully predict the efficacy of immunotherapy, urging the need for more investigations to clarify potential immune sensitization differences within polymerase epsilon/polymerase delta mutation variants.

## Introduction

Cancer stands as one of the primary causes of disease-related mortality in the world. Recent global cancer statistics show an alarming estimate of 20 million new cancer diagnoses and almost 10 million cancer-related fatalities each year, with these numbers on the increase.^1^ While conventional treatments such as surgery, chemotherapy, and radiotherapy have advanced in enhancing the overall survival rates for certain cancers, the overall effectiveness remains inadequate for the majority of cancers. Recently, immune checkpoint inhibitors (ICIs), specifically programmed cell death-1/programmed cell death-ligand 1 (PD-1/PD-L1) antibodies, have demonstrated considerable potential in treating various types of cancer such as melanoma, non-small cell lung cancer, and renal cell cancer. However, the efficacy of ICIs is relatively low in most tumor types, and varies markedly between unselected and selectively treated patients.^2^ Therefore, it is important to identify specific biomarkers to distinguish patients who could truly benefit from ICIs.

Various possible indicators for predicting the effectiveness of ICI treatment have been discovered, such as high levels of microsatellite instability/mismatch repair deficiency (MSI-H/dMMR) or high tumor mutation burden (TMB-H), as well as increased expression of PD-L1 in certain tumors.^2,3^ In a study conducted by Le et al., 53% of patients with dMMR cancers in various tumor types showed a positive response, with a complete response seen in 21% of patients.^4^ Nevertheless, the occurrence of MSI-H/dMMR is limited in clinical practice. TMB is also a predictive biomarker for the efficacy of ICI treatment in different cancers. In the phase 2 KEYNOTE-158 study, patients with TMB-H status (i.e. TMB ≥ 10 mutations per megabase) demonstrated a greater response rate than those with TMB-low (TMB-L) status (29% vs 6%, respectively).^5^ Despite the observed association between TMB and response to immunotherapy, TMB alone does not discriminate responders clearly. There are some patients with TMB-L status responding to ICIs and vice versa. Prior research has also identified additional possible predictive biomarkers, including tumor-infiltrating lymphocyte density, T-cell receptor clonality, peripheral blood markers, immune gene signatures, transcriptomic and epigenetic signatures, commensal microbiota, etc.^2,6^ Like MSI/MMR and TMB, these biomarkers have their own limitations. Hence, there is a requirement for improved biomarkers to differentiate patients who may benefit from immunotherapy and those who may not.

The Polymerase epsilon (*POLE*) and polymerase delta 1 (*POLD1*) genes are responsible for encoding the main catalytic subunit of the DNA polymerase Pol ε and Pol δ,^7^ which are involved in different proofreading and DNA repair processes such as nucleotide excision, double-strand break repair, base excision, and mismatch repair.^8^ The exonuclease region of Pol ε and Pol δ is responsible for identifying and eliminating mismatch bases formed during DNA replication. As a result, mutations in the exonuclease region of *POLE/POLD1* genes (*POL*-EDMs) lead to the loss of proofreading ability, leading to an increase in genetic mutations and the creation of neoantigens in the cell. A growing number of studies have reported that damaging germline or somatic mutations in *POLE/POLD1* genes may lead to genomic instability, heightened mutation rates, and carcinogenesis of multiple malignancies.^9–13^ Therefore, mutations of the *POLE/POLD1* (*POL*-MUTs) may enhance the sensitivity of tumors to immunotherapy and have the potential to act as a biomarker of ICI therapy.

In a previous report, our team indicated that *POL*-MUTs could be used as an independent predictive marker for the effectiveness of ICI treatment in different types of cancer.^14^ In this comprehensive analysis of a vast dataset, patients with *POL*-MUTs had a higher TMB compared to those without these mutations. Patients in the ICI treatment group who had *POL*-MUTs experienced markedly improved overall survival compared to those without these mutations (34 months vs 18 months, respectively). It should be noted that *POL*-MUTs outside of the exonuclease domain (*POL*-non-EDMs) also showed a comparable correlation with the overall survival of patients who received ICI therapy. Our further investigation revealed that domain location did not determine the predictive significance of *POL*-MUTs. Within the combined groups of 2862 cancer patients treated with immunotherapy, we discovered that both unselected *POL*-non-EDMs and *POL*-non-EDMs served as biomarkers for identifying patients who were likely to benefit from ICI therapy in addition to *POL*-EDMs. Patients with *POL*-non-EDM achieved a significantly higher response rate than the patients with wild type (58.7% vs 23.8%, respectively). *POL*-non-EDM was also linked to an immune response, even in patients with low TMB status.^15^ Despite this, there is still debate over the potential benefits of immunotherapy for patients with *POL*-non-EDMs. Some researchers suggested that tumors would only react to anti-PD-1 monotherapy if they had particular *POLE* pathogenic mutations in the DNA binding or catalytic site of the exonuclease domain.^16^ Since the majority of *POL*-MUTs were located outside the exonuclease domain, it is essential to investigate the effectiveness of ICIs in treating this patient subgroup. For this purpose, we carried out a prospective phase II clinical trial with a single arm to examine the effectiveness of toripalimab, a humanized IgG4-blocking monoclonal antibody targeting PD-1, in patients with advanced solid cancer and *POL*-MUTs who are not MSI-H.

## Results

### Patients and treatments

Between April 2019 and August 2023, a total of 15 patients diagnosed with locally advanced or metastatic colorectal adenocarcinoma, colorectal sarcoma, renal medullary carcinoma, malignant peripheral nerve sheath tumor, cervical cancer, endometrial cancer, and hepatocellular carcinoma were enrolled. All patients had confirmed germline or somatic *POL*-MUTs before enrollment. Their median age was 50 (range, 24 to 72) years. The majority of patients were diagnosed with stage IV disease and had undergone a minimum of two prior treatment regimens. Seven patients had two or more metastatic organs, and two-fifths had liver metastasis. Most of the patients (66.7%) had *POLE* mutation, and one had both *POLE* and *POLD1* mutation (Table 1 and Table 2). As of the latest follow-up, two patients had completed the two-year protocol treatment, while ten patients discontinued treatment due to disease progression, two because of grade three or higher treatment-related adverse events, and one for surgery without a radiographic assessment.

**Table 1:** Demographic and Baseline Characteristics of the Patients

Characteristic		No. of patients % (*n* = 15)
Sex	Male	5 (33.3)
	Female	10 (66.7)
Age, years	Median	50
	Range	24–72
ECOG PS	0	7 (46.7)
	1	8 (53.3)
BMI	Median	21.7
	Range	18.0–27.6
Cancer type	Colorectal adenocarcinoma	9 (60.0)
	Colorectal sarcoma	1 (6.7)
	Renal medullary carcinoma	1 (6.7)
	Malignant peripheral nerve sheath tumor	1 (6.7)
	Endometrial cancer	1 (6.7)
	Cervical cancer	1 (6.7)
	Hepatocellular carcinoma	1 (6.7)
Stage IV cancer		14 (93.3)
No. of metastasis sites	<2	8 (53.3)
	≥2	7 (46.7)
Liver metastases	Yes	6 (40.0)
	No	9 (60.0)
Previous systematic therapies	<2	4 (26.7)
	≥2	11 (73.3)
Previous resection of primary tumor		13 (86.7)
Previous radiation therapy		2 (13.3)
TMB, mut/MB	Median	6.6
	Range	1.1–203.3
Gene Mutation	*POLE*	10 (66.7)
	*POLD1*	4 (26.7)
	*POLE* & *POLD1*	1 (6.7)

**Table 2:** Individual patient data

No.	Sex, age	Cancer type	TMB	Response	PFS (months)	OS (months)	*POLE/POLD1* mutation	Mutation alteration	cDNA change	Variant type	POL-EDM	Functional mutation	OncoKB curated alterations
SYSUCC01	F, 55 yr	HCC	4.6	SD	4.9	6.1	*POLE*	p.H1440T	c.4318 C > T	missense	N	VUS	Unknown
SYSUCC02	F, 65 yr	EC	1.1	SD	2.6	28.2	*POLE*	p.A252V (germline)	c.755 C > T	missense	N	VUS	Unknown
SYSUCC03	F, 58 yr	CRC	2.8	PD	1.4	53.0	*POLE*	p.V533M	c.1597 G > A	missense	N	VUS	Unknown
SYSUCC04	F, 24 yr	malignant peripheral nerve sheath tumor	25.1	NE	NA	17.9	*POLE*	p.I2070-K2072delIQK	c.6209-6217delTTCAGAAGA	DEL	N	VUS	Unknown
							*POLE*	p.T2273-E2275delTLE	c.6817-6825delACCCTGGAG	DEL	N	VUS	Unknown
							*POLD1*	p.L523-L526delLERL	c.1566-1577del12	DEL	Y	VUS	Unknown
SYSUCC05	M, 41 yr	CRC	2.6	SD	2.7	38.7	*POLE*	p.T1104M	c.3311 C > T	missense	N	VUS	Unknown
SYSUCC06	M, 52 yr	CRC	5.3	PD	1.2	13.5	*POLD1*	p.T238M	c.713 C > T	missense	N	VUS	Unknown
SYSUCC07	F, 64 yr	Renal medullary carcinoma	2.6	PR	39.9	40.2	*POLD1*	p.R682Q (germline)	c.2045 G > A	missense	N	VUS	Unknown
SYSUCC08	F, 47 yr	CRC	92.8	PD	1.5	22.5	*POLE*	p.V411L	c.1231 G > T	missense	Y	Functional Mutation/ Hypermutation	Likely Oncogenic
SYSUCC09	F, 35 yr	CRC	130.3	PR	24.8	39.4	*POLE*	p.S297F	c.890 C > T	missense	Y	Functional Mutation/ Hypermutation	Likely Oncogenic
								p.S1906Y	c.5717 C > A	missense	N	VUS	Unknown
SYSUCC10	M, 45 yr	CRC	203.3	CR	25.0	33.4	*POLE*	p.P286R	c.857 C > G	missense	Y	Functional Mutation/ Hypermutation	Likely Oncogenic
								p.F1907L	c.5721 C > A	missense	N	VUS	Unknown
SYSUCC11	F, 72 yr	Cervical cancer	20.2	PD	1.3	17.5	*POLD1*	p.S1034F	c.3101 C > T	missense	N	VUS	Unknown
SYSUCC12	F, 50 yr	CRC	5.3	SD	2.6	14.4	*POLE*	p.Q520P	c.1559 A > C	missense	N	VUS	Unknown
SYSUCC13	F, 48 yr	CRC	16.3	PD	1.4	5.0	*POLE*	p.E575K	c.1723 G > A	missense	N	VUS	Unknown
SYSUCC14	M, 56 yr	CRC	6.6	PD	0.4	0.4	*POLD1*	p.R978C	c.2933 C > T	missense	N	VUS	Unknown
SYSUCC15	M, 39 yr	CRC	6.7	SD	2.5	8.7	*POLE*	p.I1739V	c.5215 A > G	missense	N	VUS	Unknown

*cDNA* complementary DNA, *CR* complete response, *CRC* colorectal cancer, *EC* endometrial cancer, *F* female, *HCC* hepatocellular carcinoma, *M* male, *N* no, *OS* overall survival, *PFS* progression-free survival, *POL-EDM* *POLE/POLD1* exonuclease domain mutation, *PR* partial response, *SD* stable disease, *TMB-H* high tumor mutational burden, *TMB-L* low tumor mutational burden, *VUS* variant of unknown significance, *Y* yes

### Anti-tumor activity

**Fig. 1:**
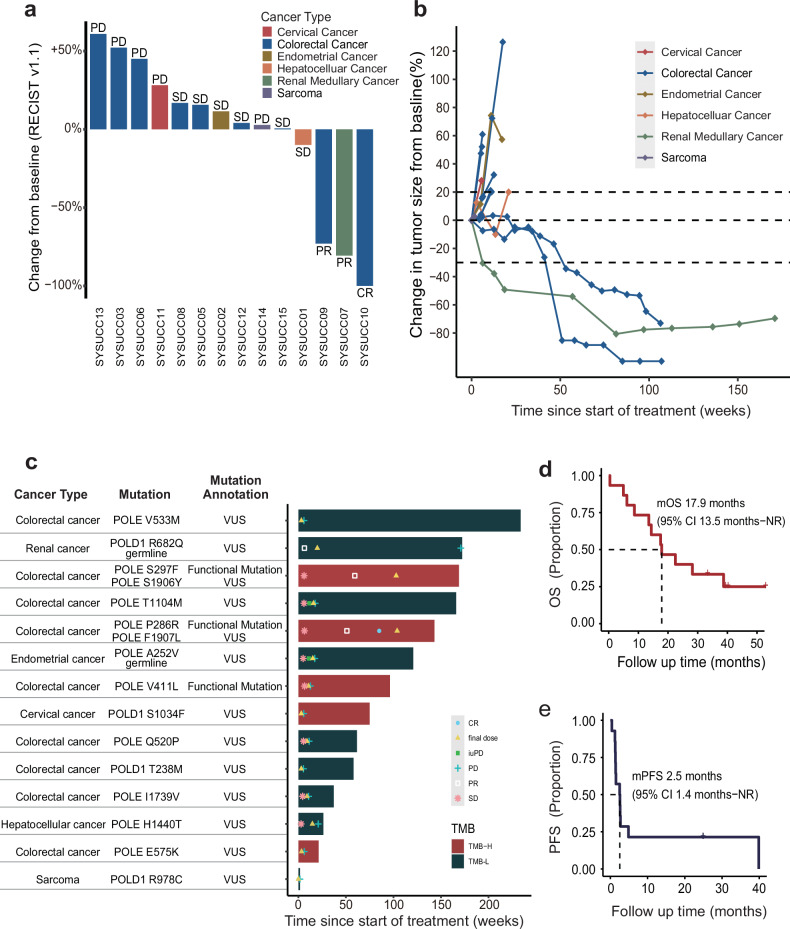
Tumor response assessment and Kaplan-Meier survival plots. **a** Waterfall plot depicting maximum changes in tumor size from baseline of the 14 evaluated patients. **b** Spider plot depicting longitudinal changes in individual tumor measurements over time for the 14 evaluated patients. **c** Swimmer plot depicting treatment exposure, clinical response and survival of the 14 evaluated patients. **d** Kaplan-Meier plot of overall survival of the 15 investigated patients. **e** Kaplan-Meier plot of the progression-free survival of the 15 patients

Response to toripalimab was evaluated in 14 patients who underwent post-baseline assessment. We observed an objective response in three patients (one complete response and two partial responses), and five patients had stable disease (Fig. 1 and Table 2). The overall response rate was 21.4% with a confidence interval of 95% between 4.7% and 50.8%, and the disease control rate was 57.1% with a confidence interval of 95% between 28.9% and 82.3%. Among the three responders, two patients demonstrated both an EDM and a non-EDM *POLE* mutation, with one showing a complete response and the other a partial response. The third patient (a partial response) had a germline non-EDM *POLD1* mutation. The study could advance to the second stage if there were at least 3 out of 18 cases showing objective responses in the first stage, leading to the enrollment of an additional 17 patients. Despite the high disease control rate of 54.5% in patients with *POL*-non-EDMs, the overall response rate was only 9.1%. These results indicated that this group of patients needs more intensive treatment. Following conversations with the data monitoring committee and researchers, it was determined that the initial findings of the ongoing study would be disclosed, and a new clinical trial would be organized to investigate the effectiveness of combining immunotherapy with chemotherapy in patients with *POL*-non-EDMs.

**Fig. 2:**
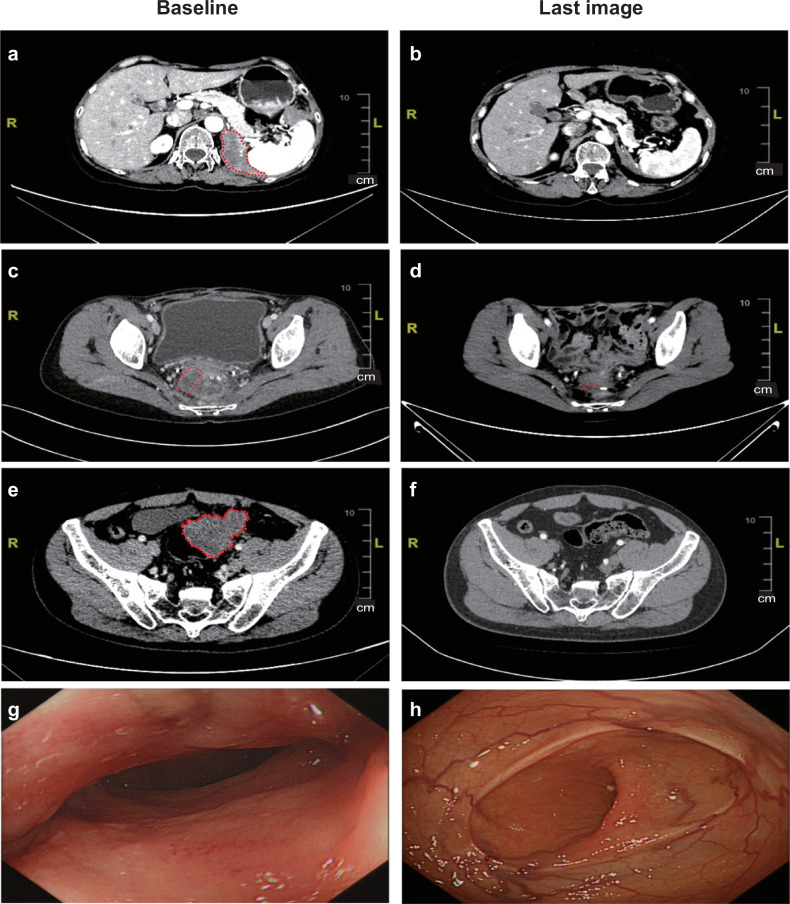
Objective tumor responses in three patients with metastatic solid tumor. CT scan showing peritoneal metastasis and its evolution between baseline (**a**) and after toripalimab (**b**) of patient SYSUCC07 with metastatic renal medullary carcinoma harboring R682Q germline *POLD1* mutation. **c**, **d** The regression of the peritoneal metastasis of patient SYSUCC09 diagnosed with colon cancer harboring S297F and S1906Y *POLE* mutation. **e**, **f** The locally recurrent primary tumor and its evolution between pretreatment and after toripalimab in patient SYSUCC10 diagnosed with colon cancer harboring P286R and F1907L *POLE* mutation, who achieved complete response. **g**, **h** The colonoscopy image at baseline and after treatment of patient SYSUCC10, respectively

Figure 1a–c present the overall results stratified by TMB, cancer type, and *POL* functional mutation/hypermutation.^17^ The median overall survival was 17.9 months, with a 95% confidence interval of 13.5 to not reached (NR), as shown in Fig. 1d. The median progression-free survival was 2.5 months, with a 95% confidence interval of 1.4 to NR, as shown in Fig. 1e. Figure 2 illustrates the baseline and post-treatment examinations of three patients with objective tumor responses. Patient SYSUCC07, diagnosed with R682Q *POLD1* germline mutant renal medullary carcinoma, achieved partial response after two doses of toripalimab. Despite discontinuing treatment after seven cycles due to grade three treatment-related pneumonitis, the patient continued to exhibit a persisting partial response with a progression-free survival of 39.9 months (Fig. 2a, b). Patient SYSUCC09, who had local recurrent colon cancer with S297F and S1906Y *POLE* mutations and had previously received two lines of palliative systemic treatment, also achieved a partial response after 18 doses of toripalimab. She has completed 2-year treatments with a persisting partial response at the last follow-up (Fig. 2c, d). One patient (SYSUCC10) achieved a complete response after receiving protocol treatment. This 45-year-old male had local recurrent colon cancer and liver metastasis with P286R and F1907L *POLE* mutations. After standard palliative chemotherapy, he was enrolled in this clinical trial. He achieved a partial response after receiving 14 doses of toripalimab and a complete response after 26 cycles of treatment, with both coloscopy and PET/CT scan confirming no tumor residue. At the last follow-up, he had completed two-year treatments and achieved a confirmed complete response (Fig. 2e–h).

Patients SYSUCC02 and SYSUCC06 were assessed with progressive diseases during the second and first radiological assessments, respectively. However, they were both clinically stable with preserved performance status. After thorough evaluation, investigators considered that the patients may continue to benefit from the study treatment. Both patients, who were subsequently reassessed as having stable disease, received additional doses of toripalimab before disease progression—SYSUCC02 received four more doses, and SYSUCC06 received eight more doses.

### Treatment-related toxicities

Among the 15 patients, nine (60.0%) reported at least one treatment-related adverse event: three (20.0%) experienced grade three or higher adverse events, and one (6.7%) experienced grade five adverse events. One patient with colorectal sarcoma and liver metastasis died from hepatic hemorrhage and hepatic failure after one cycle of the protocol therapy, which was believed to be related to the treatment. The most common treatment-related adverse events included elevated transaminase (33.3%), anemia (20.0%), and hypothyroidism (20.0%). Grade three treatment-related adverse events comprised hepatic hemorrhage, hepatic failure, anorexia, diarrhea, and pneumonitis (Table 3).

**Table 3:** Summary of treatment-related adverse events

Adverse events		All Grades (%)	Grade 3-5 (%)
Blood or lymphatic system disorders	Anemia	3 (20.0%)	0 (0.0%)
	Leukopenia	1 (6.7%)	0 (0.0%)
Gastrointestinal disorders	Anorexia	1 (6.7%)	1 (6.7%)
	Diarrhea	1 (6.7%)	1 (6.7%)
Hepatobiliary disorders	Elevated transaminase	5 (33.3%)	0 (0.0%)
	Hepatic failure	1 (6.7%)	1 (6.7%)
	Hepatic hemorrhage	1 (6.7%)	1 (6.7%)
Endocrine disorders	Hypothyroidism	3 (20.0%)	0 (0.0%)
Pulmonary disorders	Pneumonitis	1 (6.7%)	1 (6.7%)
Renal and urinary disorders	Proteinuria	2 (13.3%)	0 (0.0%)
Fatigue		2 (13.3%)	0 (0.0%)

### *POLE/POLD1* mutation and treatment response

**Fig. 3:**
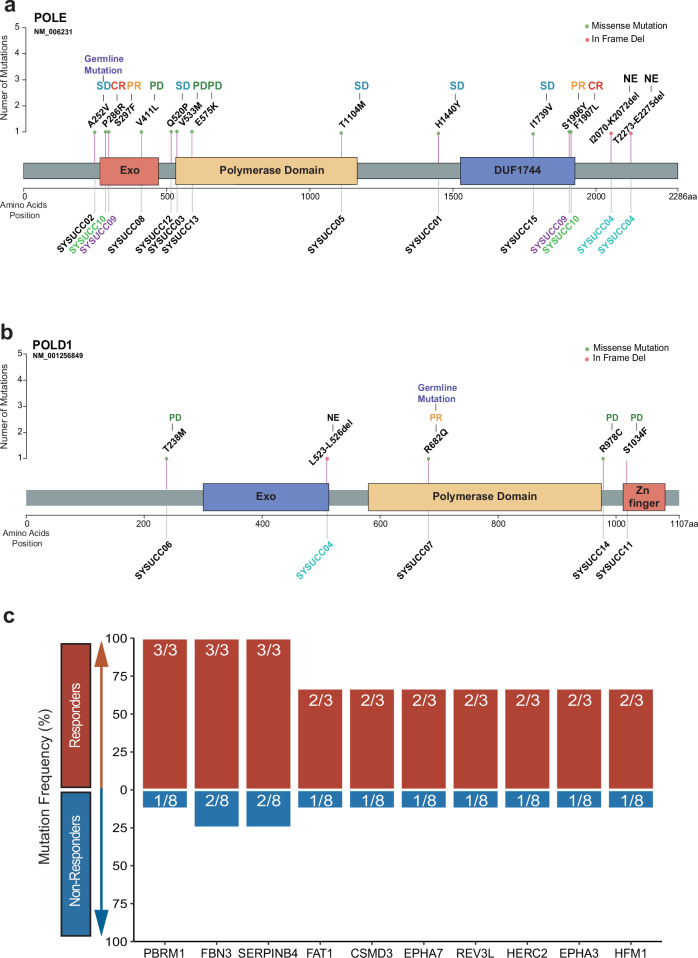
*POLE/POLD1* Variant and Co-mutation Gene. **a**, **b** Individual responses for each *POLE/POLD1* variant. CR complete response, NE not evaluated, PR partial response, SD stable disease, PD progressive disease. **c** Mutation frequency of top 10 genes with the greatest mutation difference between responders and non-responders. The upper panel displays the mutation frequency among responders, and the lower panel presents the same for non-responders. The fraction in the bar indicates the specific number of patients carrying the mutation among responders or non-responders. Only *PBRM1* enriched in responders (Fisher’s Exact Test *P* < 0.05)

*POLE/POLD1* variants are detailed in Table 2 and Fig. 3. Of all 15 patients, ten had *POLE* mutation (one A252V variant was germline), four had *POLD1* mutation (one R682Q variant was germline), and one had malignant peripheral nerve sheath tumor with both *POLE* and *POLD1* mutations. Most patients had one *POLE* or *POLD1* mutation, while three had more than one mutation: one with S297F and S1906Y *POLE* mutations, one with P286R and F1907L *POLE* mutations, one with L523-L526del *POLD1* mutation, I2070-K2072del and T2273-E2275del *POLE* mutations. Among three patients with more than one mutation, two had an objective response, and one did not undergo a CT scan after one dose of toripalimab.

Both *POL*-EDMs and *POL*-non-EDMs might obtain clinical benefits from immunotherapy. Patients with *POL*-EDMs had an overall response rate of 66.7% and a disease control rate of 66.7%, compared to 9.1% and 54.5% for *POL*-non-EDMs, respectively. Of all 15 patients, 12 had *POL*-non-EDMs, while four had *POL*-EDMs, including one with P286R *POLE* mutation, one with V411L *POLE* mutation, one with S297F *POLE* mutation, and one with L523-L526del *POLD1* mutation. Three patients with *POLE*-EDMs were likely oncogenic (annotated by OncoKB: https://www.oncokb.org/) or functional mutation/hypermutation, two of them (66.7%) achieved an objective response (SYSUCC09 with PR and SYSUCC 10 with CR), while all *POL*-non-EDMs were variants of unknown significance (VUS).

*POL*-related mutation signatures may reflect *POL* functional mutation status more accurately than *POL* point mutation. Ma X et al. constructed a model based on 4 *POL*-MUT signatures to identify tumors with functional *POL*-MUT sensitive to ICB.^17^ The 4 *POL*-MUT signatures include SBS10a, SBS10b, SBS20 and SBS14. To investigate the role of *POL*-MUT signatures in our trial, we compared the attribution of *POL*-MUT signatures between responders and non-responders. We found the mutations of all responders are dominated by *POL*-MUT signatures: *POL*-MUT signatures attributed to 55%, 40%, and 65% mutations in SYSUCC10, SYSUCC07, and SYSUCC09, respectively (Supplementary Fig. 1). As for the non-responders, most of them were dominated by other non-*POLE/POLD1* mutation-related signatures (e.g. SBS30). Only one patient with stable disease was dominated by *POL*-MUT signatures. Surprisingly, in one patient with partial response (SYSUCC09), *POL*-MUT signatures were eliminated after immunotherapy. These results indicated that the *POL*-MUT signatures might be favorable predictive factors for immunotherapy.

### Patients with *POLE/POLD1* and *PBRM1* co-mutation respond better to immunotherapy

To delineate the profile of patients who responded to immunotherapy, we focused on genomic differences between responders and non-responders. Notably, four patients harbored *PBRM1* gene mutation, and three of them responded to immunotherapy (Fisher’s Exact Test *P* = 0.024) (Fig. 3c). Acknowledging the scarcity of patients with concurrent *POL*-MUTs and *PBRM1* mutations in publicly available immunotherapy cohorts, we explored the role of *POL* and *PBRM1* co-mutation in two large pan-cancer cohorts: the MSKCC metastasis cancer cohort^18^ and the TCGA pan-cancer cohort.^19^ All patients in these cohorts were classified into four groups: both *POL* and *PBRM1* wild type (WT), only *POL*-MUT (*POL*-o), only *PRBM1* (*PBRM1*-o) and *POL-PBRM1* co-mutation (*POL*&*PBRM1*) respectively. In the MSKCC cohort, after removing MSI-H patients and adjusting age, gender, and cancer types, the *POL*&*PBRM1* group had the highest TMB value (*P* < 0.001) (Supplementary Fig. 2a, Supplementary Table 1).

Given the profound influence of the mutation site in *POL* on TMB, patients with *POL*-MUTs were stratified by the mutations in EDM and non-EDM. Supplementary Fig. 2b showed that patients with *PBRM1*&*POL* also harbored much higher TMB than those with *POL*-o regardless of the mutation location (EDM: *P* < 0.001, non-EDM: *P* < 0.001) (Supplementary Table 1). In the three cancer types with the most *POL*&*PBRM1* patients (colorectal cancer, endometrial cancer, and non-small cell lung cancer), the *POL*&*PBRM1* group also had the highest TMB among the four groups (Supplementary Fig. 2c). Higher TMB in these three cancer types with *POL*&*PBRM1* mutation was observed both in the EDM group and non-EDM group compared to those with POL-o (Supplementary Fig. 2d). Similar results were also observed in the TCGA pan-cancer cohort (Supplementary Fig. 3a–d, Supplementary Table 2). To validate the role of *POL*&*PBRM1* in immunotherapy further, we evaluated six transcriptional immunotherapy response signatures (T cell-inflamed gene expression profiles (T Cell GEP) signature,^20^ Tertiary lymphoid structures (TLS) signature,^21^ interferon-gamma (IFNG) signature,^22^ Tumor Inflammation Signature (TIS),^23^ Immunologic Constant of Rejection (ICR) signature^24^ and cytotoxic T lymphocytes (CTL) signature^22^) in TCGA. After adjusting for cancer types and filtering out MSI-High samples, the *POL*&*PBRM1* group had the highest signature scores in all response signatures (GEP: FDR < 0.001, IFG: FDR < 0.001, TLS: FDR = 0.088, TIS: FDR < 0.001, ICR: FDR < 0.001, CTL: FDR < 0.001) (Supplementary Fig. 4a, Supplementary Table 3). Next, we investigated the pathway and tumor microenvironment differences between *POL*&*PBRM1* and others. GSEA showed that immune-related-pathways, including the TCR signaling pathway, chemokine receptors bind chemokines, interferon gamma signaling, and antigen processing−cross presentation, were significantly activated in the *POL*&*PBRM1* group (Supplementary Fig. 4d–f). DNA repair and DNA double-strand break response were suppressed in the *POL*&*PBRM1* group. Given the significant heterogeneity of tumor microenvironment among various types of cancers, we focused on two cancer types with the most *POL*&*PBRM1* patients: colorectal cancer and endometrial cancer. In colorectal cancer, the *POL*&*PBRM1* group had more CD4^+^ memory T cells, and CD8^+^ T cells (CD8^+^ naive T cells, CD8^+^ effector memory T cells, and CD8^+^ central memory T cells) (Supplementary Fig. 4b). Similarly, more CD4^+^ T cells and CD8^+^ T cells were also observed in the *POL*&*PBRM1* group in endometrial cancer (Supplementary Fig. 4c). In summary, our findings revealed that patients with *POL*&*PBRM1* co-mutations exhibited significantly higher TMB, higher immunotherapy response signature scores, and more active anti-tumor immunity, suggesting a potentially favorable response to immunotherapy in these patients.

### Disease progression accompanied by the increase of *POL* VAF

Previous studies^25–27^ have established that ctDNA monitoring serves as a potent instrument for treatment management, in which an increase in ctDNA levels during therapy indicates disease progression. Therefore, it is worthwhile to track the ctDNA dynamic change during treatment. In our cohort, four patients underwent ctDNA monitoring at more than three-time points (Supplementary Fig. 5, Supplementary Table 4). A dynamic change in *POL* maximal variant allele frequency (*POL* VAF) was consistent with tumor burden and treatment response for the patients with somatic *POL-*MUTs. In two responders (SYSUCC09 and SYSUCC10), *POL* VAF was detected at an extremely low level before treatment (0.63% for SYSUCC09 and 0.52% for SYSUCC10), and it remained undetectable throughout the clinical response. For patient SYSUCC06, the *POL* VAF remained at an almost undetectable level before the second dose of treatment, but then dramatically increased from 4.1% to 25.3% when the volume of the tumor increased, following which the patient was confirmed with progressive disease after ten doses of treatment. The *POL* VAF of patient SYSUCC07, who achieved partial response, did not change because the tumor harbored a *POLD1* germline mutation. According to the series ctDNA analysis, increasing ctDNA *POL* VAF levels may provide moderated valuable insights into the progression of the tumor in patients with *POL-*MUTs.

## Discussion

This prospective clinical trial evaluated the anti-tumor effect of toripalimab in non-MSI-H patients with unselective *POLE*/*POLD1* mutations. Three patients achieved an objective response, meeting the primary endpoint of the first stage with an overall response rate of 21.4%.

The findings indicated that patients with solid tumors plus *POL*-EDMs who received toripalimab exhibited a significant response rate (66.7%), consistent with previous studies.^16,28^ As *POL*-EDMs only account for a small proportion of all *POL*-MUTs, it is important to explore the predictive significance of *POL*-non-EDMs in immunotherapy. The recently reported Acsé Nivolumab trial focused on patients with advanced *POLE*-mutated solid tumors with mismatch repair-proficient phenotype. The study showed that monotherapy with nivolumab achieved a 38% overall response rate and 58% disease control rate at 12 weeks.^16^ Researchers suggested that immunotherapy could be advantageous exclusively for patients with variants impacting the proofreading function, DNA binding clusters, or the catalytic site, while no anti-tumor activity was noted in patients possessing *POLE* non-exonuclease domain mutations.^16^ However, our previous study discovered that both unselected *POL*-MUTs and *POL*-non-EDMs could act as biomarkers for identifying responders to immunotherapy.^15^ Notably, *POL*-non-EDMs were linked to an activated immune response, even in low-TMB tumors. This finding indicates that certain *POL*-MUTs might elicit an immune response through mechanisms not dependent on hypermutant status.^15^ In another study at the MD Anderson Cancer Center,^28^ 82 patients who were diagnosed with *POLE* mutant advanced solid tumors were treated with PD-1/PD-L1 inhibitor with or without a CTLA-4 inhibitor, resulting in a 35% overall response rate and 60% disease control rate. Further analysis of the impact of *POLE* mutation locations revealed that patients with mutations in both the inside and outside of the exonuclease domain could benefit from ICI therapy (overall response rate, 33 and 36%; disease control rate, 78 and 58%, respectively). Dong et al. analyzed the MSK-IMPACT cohort of 1278 patients who were diagnosed with advanced cancers harboring low or intermediate TMB and received ICIs. The study showed that missense *POLE* mutations outside the exonuclease domain were predictive markers of ICI benefit.^29^ In the current study, anti-PD-1 therapy was not exclusively ineffective among patients with *POL*-non-EDMs, though the response rate was relatively low. One patient with *POL*-non-EDM mutation achieved PR and had a PFS over 3 years, and one patient achieved disease control and had an approximately 5-month PFS, suggesting that some patients could still derive clinical benefits from ICI therapy. In addition, one patient with *POLE*-EDM and high TMB showed no response to PD-1 antibody and progressed quickly. Further investigation is needed to better classify this subgroup and identify patients who could truly benefit from immunotherapy.

This study revealed that *POL*-EDM or non-EDM status alone cannot fully predict the benefit of ICI treatment, highlighting the need for additional biomarkers. We found that patients carrying *PBRM1* mutation were enriched in responders. In pan-cancer cohorts, we discovered that patients carrying *POL &* *PBRM1* mutations may have better responses to ICIs. Previous studies showed patients with truncating or loss-of-function mutations of *PBRM1* benefited from ICI.^30,31^ BAF180, encoded by *PBRM1*, is a submodule of the PBAF, playing a key tumor suppressor role by regulating cellular differentiation, proliferation, and DNA repair.^32,33^ Mutation of BAF180 affects DNA repair in three ways: increasing the distance of centromeres, impairing the DNA post-replication repair pathway, and losing function to silence transcription near the site of damage.^34,35^ In addition to increasing TMB, Pan et al. discovered that *PBRM1* expression in tumor cells negatively correlated with T-cell cytotoxicity markers.^36^ Loss of PBAF function increased tumor cell sensitivity to interferon-γ and *PBRM1*-deficient tumor triggered T cell-mediated killing more easily by activating interferon-γ than those with intact *PBRM1*.^36^ If patients carried *PBRM1* and *POL*-MUTs simultaneously, massive numbers of mutations would be produced and could not be cleared in time because of the synergistic effect on the DNA repair system, leading to increasing TMB. At the same time, *PBRM1* mutation would up-regulate immune-related pathways and recruit more effector T cells. Therefore, patients with *PBRM1* and *POL* co-mutation may respond better to ICIs. Moreover, the synergistic effect of *POL &* *PBRM1* mutation has sparked inspiration for a novel therapeutic strategy for patients with *POL*-MUTs: inhibiting key molecules of the DNA repair system, such as *PBRM1*, will further increase TMB, which holds the potential to enhance the effectiveness of immunotherapy.

Some limitations of this study should be acknowledged. First, the sample size was relatively small due to the low prevalence of *POL*-MUTs. Although the preliminary results showed the potential clinical benefits of ICI, more data from prospective studies are needed to confirm these findings. Second, selection bias might have existed as most of the cancers in this study were colorectal cancers. Third, blood samples were missing from several treatment cycles for some patients, and the treatment duration of some patients was too short due to disease progression, thereby affecting the assessment of ctDNA change during therapy. More ctDNA data is needed to validate the value of ctDNA *POL* VAF in clinical benefit prediction.

In summary, this study demonstrates that anti-PD-1 therapy elicited a favorable objective response in *POLE/POLD1* exonuclease domain mutant tumors and a good disease control rate in *POLE/POLD1* non-exonuclease domain mutant tumors, indicating the need to explore the efficacy of immunotherapy combined with chemotherapy in patients with *POL*-non-EDMs. Further classification of the *POL*-non-EDMs to identify the potential subgroups sensitive to immunotherapy is also a valuable area for investigation.

## Materials and methods

### Study design and patients

Investigators initiated a phase II clinical trial at Sun Yat-sen University Cancer Center in Guangzhou, China, and involved a single arm with an open-label design. The study criteria included patients between the ages of 18 and 75 with pathologically confirmed metastatic or unresectable solid tumors, microsatellite stability (MSS), microsatellite instability-low (MSI-L), or proficient mismatch repair (pMMR), germline or somatic *POLE/POLD1* mutation (excluding synonymous mutation), refractory or intolerant to systemic chemotherapy or target therapy, adequate organ function, and an Eastern Cooperative Oncology Group (ECOG) performance status of 2 or less. Major exclusion criteria included prior use of PD-1 antibody or other immune checkpoint inhibitors, presence of suspected or confirmed brain metastasis, history of another cancer, autoimmune disease, or long-term immunosuppressant use, as well as HIV infection, active hepatitis, severe infections needing systemic antibiotics, or unexplained fever. Although Shanghai Junshi Biosciences donated the study drug, they did not participate in any aspect of data collection or analysis.

The study protocol was approved by the institutional review board (B2018-153-03) and conducted in accordance with the Declaration of Helsinki and the Good Clinical Practice guidelines. All patients provided written informed consent for participation before enrollment. This study has been registered on “https://clinicaltrials.gov/” (identifier: NCT03810339).

### Procedures

After enrollment, the patients received 240 mg of toripalimab through intravenous infusion every three weeks, with evaluations of their response every 6 weeks. At baseline and every six weeks until disease progression, computed tomography or magnetic resonance imaging was performed. Patients who had received at least one cycle of toripalimab and had undergone at least one post-baseline assessment were assessed for tumor response based on Response Evaluation Criteria in Solid Tumors version 1.1 (RECIST 1.1). Patients with disease control were eligible to continue treatment for a maximum of two years unless they experienced intolerable adverse events, confirmed progressive disease or withdrew consent. For patients assessed as pseudoprogression, treatment would continue until confirmed progressive disease.

Patients had safety monitoring visits at the end of each cycle, with follow-up visits at 30 days and 6 months after the last dose. Dose delays were allowed to manage grade three or four adverse events (National Cancer Institute Common Terminology Criteria for Adverse Events [NCI-CTCAE; version 5.0]). Dose reductions or dose escalations were not permitted.

Tumor tissue and plasma samples were collected for genomics and ctDNA analysis. Tissue samples preserved in formalin and embedded in paraffin were gathered for DNA sequencing of tumors with a 680-gene next-generation sequencing (NGS) panel that is commercially accessible.

### Outcomes

The primary endpoint of the research was the overall response rate, which was defined as the percentage of patients who achieved either a complete response or partial response based on RECIST 1.1. Additional outcome measures consisted of disease control rate, overall survival, progression-free survival, and safety assessments. The disease control rate was determined by calculating the percentage of patients who achieved complete response, partial response, or stable disease as their best overall response. Overall survival was defined as the period from initial toripalimab administration until death, while progression-free survival was determined as the period from initial toripalimab administration until the occurrence of progressive disease or death, whichever came first. Adverse events were assessed for severity in patients who underwent at least one treatment cycle using NCI-CTCAE version 5.0.

### DNA extraction and library construction

All tissue and blood samples were acquired with informed consent. In total, 56 samples of patients at baseline, post-treatment, and after disease progression were obtained for tissue DNA or circulating tumor DNA (ctDNA) analysis.

Blood samples were centrifuged within two hours of collection to isolate peripheral blood lymphocyte (PBL) debris and plasma. Next, circulating cell-free DNA (cfDNA) was isolated from the plasma with the QIAamp Circulating Nucleic Acid kit (QIAGEN), while genomic DNA (gDNA) was extracted from corresponding PBLs using the RelaxGene Blood DNA System (TianGen Biotech Co., Ltd., China). Tumor DNA was isolated from tumor tissue samples preserved in formalin and embedded in paraffin using the QIAamp DNA FFPE tissue kit from Qiagen. Extracted DNA was measured using Qubit 2.0 from Thermo Fisher Scientific in the USA in accordance with the manufacturer’s instructions.

Initially, FFPE tumor tissue DNA and genomic DNA (gDNA) underwent fragmentation via enzymatic digestion using dsDNA Fragmentase from NEB. Subsequently, size selection of the DNA fragments (150–250 bp) was achieved utilizing Ampure XP beads (Beckman Coulter, Inc., Brea, CA, USA). The KAPA Library Preparation kit from Kapa Biosystems, Inc. in Wilmington, MA, USA, was used to create sequence libraries. This involved checking the DNA fragment concentration, repairing the ends, adding A-tails to the 3’-end, and performing PCR amplification following the manufacturer’s instructions.

### Targeted capture and sequencing

Targeted capture was executed utilizing DNA probes specifically designed to encompass all exons and select intron regions across 680 cancer-related genes, in addition to 117 microsatellite instability sites. The hybridization of the amplified sample libraries and the SeqCap EZ Library was used following the manufacturer’s instructions. Following the hybrid selection process, the DNA fragments that were captured underwent amplification with 1× KAPA HiFi Hot Start Ready Mix and Post-LM-PCR Oligos. Afterward, the two reactions were then pooled and purified using Agencourt AMPure XP beads. Next, the libraries underwent sequencing with 150 bp paired-end reads on the Illumina NovaSeq 6000 platform (Illumina).

### Raw data processing and alignment

Raw sequencing data were pre-processed by Fastp (version 0.18.0), which encompassed adaptor trimming, meticulous removal of low-quality and short reads to ensure data integrity.^37^ The resulting clean reads were then precisely aligned to the human reference genome hg19 genome (GRch37) using the Burrows-Wheeler Aligner v0.7.15 r1140 with default settings.^38^ Subsequently, Gencore (version 0.12.0) was employed to eliminate duplicate reads, streamlining the dataset. Finally, Samtools (version 0.1.19) was utilized to generate mpileup files, focusing solely on paired reads with a mapping quality score ≥ 60, ensuring high confidence in variant calling.^39,40^

### Mutation calling, filtering and annotation

VarScan2 (version 2.3.8) was utilized to identify single nucleotide variants (SNVs) and short insertions/deletions (indels).^41^ After deduplication, the average sequencing depth achieved for cfDNA and tumor tissue DNA were ≥500× and ≥1500×, respectively. For cfDNA, stringent filter criteria were applied, including a variant allele frequency (VAF) threshold of 0.1%, requiring at least five unique reads with at least one on each strand for somatic variants (SNVs or indels), and exclusion of variants with a mutant allelic frequency greater than 0.5% in the paired normal sample (PBLs). Furthermore, cfDNA SNVs and indels were subjected to background polishing using healthy subject cfDNA samples to minimize false positives. For tumor tissue DNA, the filter criteria were adjusted with a VAF threshold of 2%, maintaining the requirement for at least five unique reads, with at least one read present on each strand, and <0.5% mutant allelic frequency in PBLs. An additional layer of quality control involved manual visual inspection using GenomeBrowse to eliminate potential artifacts. Subsequently, ANNOVAR (version 2018-04-16) was employed to annotate all identified SNVs and indels.^42^ Blood tumor mutational burden (bTMB) was estimated from ctDNA by calculating the total number of SNVs and indels with a VAF exceeding 0.5% per million bases. Tumor mutational burden (TMB) was estimated for tumor tissue DNA based on SNVs and indels with a VAF above 5% per million bases. MSIsensor2 (https://github.com/niu-lab/msisensor2) was applied to assess the microsatellite instability (MSI) status.

### Mutation signature analysis

Additional 14 tumor samples from the 15 patients were collected for NGS, among which 11 were obtained before treatment, and three were obtained at the end of treatment. One sample with less than 10 mutations was filtered.^17^ The mutation data were transformed into a trinucleotide context matrix and then normalized, taking into account the relative abundance of each trinucleotide context category within the HaploX gene panel. The normalized trinucleotide context matrices were refitted to 60 COSMIC V3 Single Base Signature (SBS) using the deconstructSigs R package. The SBS signatures with no contribution to all samples were removed, and the contributions of the rest SBS signatures were shown by the pheatmap R package.

### External cohort collection

Mutation data of MSKCC metastasis cancer cohort^18^ were obtained from cBioPortal (https://www.cbioportal.org/datasets). TCGA pan-cancer mutation data and RNA-sequencing data were downloaded from Xena (https://xenabrowser.net/).^19^

### TMB, survival, and transcriptome analysis

We utilized a standard generalized linear regression model, incorporating age, gender, and cancer type as covariates, to investigate the relationship between TMB and gene mutation groups. T-test was applied to compare the TMB value in groups. The R packages “survival” and “survminer” were used to perform survival analysis. The multivariate Cox-proportional hazard modeling was utilized to estimate the hazard ratio among gene mutation groups. Cancer type, age, and gender were set as the covariates in the model. The immune response signature scores were estimated by ssGSEA in the GSVA R package. A generalized linear regression model, similar to TMB, was built to validate the relation between these signature scores and gene mutation groups. Different gene expression analysis was performed by DESeq2. We performed GSEA analysis on the different gene expression results obtained from DESeq2 based on the clusterProfiler R package. Tumor microenvironment was estimated by xCell.

### Quantification and statistical analysis

Simon’s two-stage design was chosen to calculate sample size with *P*_0_ = 0.1 (as the null hypothesis) and *P*_*1*_ = 0.3 (as the alternative hypothesis), at a significance level of 0.05 and a power of 90%. In the first stage, 18 patients were needed, and the study had to stop if two or fewer patients achieved an objective response. If three or more patients demonstrated an objective response, another 17 patients were to be recruited in the second stage. If more than six of these 35 patients achieved an objective response, the regimen was considered effective.

Overall survival and progression-free survival were analyzed using the Kaplan–Meier method and the stratified Log-rank test to estimate medians and 95% CIs. The overall response rate and disease control rate were calculated using the exact Clopper-Pearson CIs. Statistical analyses were performed using the R 4.0.2 software.

### Supplementary information

Study Protocol

Supplementary file

Informed Consent Form

## Data Availability

The unprocessed sequence information mentioned in this article has been stored in the Genome Sequence Archive^43^ at the National Genomics Data Center,^44^ China National Center for Bioinformation / Beijing Institute of Genomics, Chinese Academy of Sciences (GSA-Human: HRA007914), which can be accessed by the public at https://ngdc.cncb.ac.cn/gsa-human. The analysis code can be found at https://github.com/runjie-huang/POL_Mut. For further details needed to reassess the data presented in the article, the lead contact can provide upon reasonable request.

## References

[1] Sung, H. et al. Global cancer statistics 2020: GLOBOCAN estimates of incidence and mortality worldwide for 36 cancers in 185 countries. *CA Cancer J. Clin.* **71**, 209–249 (2021). 33538338 10.3322/caac.21660

[2] Gibney, G. T., Weiner, L. M. & Atkins, M. B. Predictive biomarkers for checkpoint inhibitor-based immunotherapy. *Lancet Oncol.* **17**, e542–e551 (2016). 27924752 10.1016/S1470-2045(16)30406-5 PMC5702534

[3] Zhou, C. et al. Outcomes and toxicities of immune checkpoint inhibitors in colorectal cancer: a real-world retrospective analysis. *Cancer Commun. (Lond.)* **41**, 921–924 (2021). 34327863 10.1002/cac2.12199 PMC8441054

[4] Le, D. T. et al. Mismatch repair deficiency predicts response of solid tumors to PD-1 blockade. *Science* **357**, 409–413 (2017). 28596308 10.1126/science.aan6733 PMC5576142

[5] Marabelle, A. et al. Association of tumour mutational burden with outcomes in patients with advanced solid tumours treated with pembrolizumab: prospective biomarker analysis of the multicohort, open-label, phase 2 KEYNOTE-158 study. *Lancet Oncol.* **21**, 1353–1365 (2020). 32919526 10.1016/S1470-2045(20)30445-9

[6] Havel, J. J., Chowell, D. & Chan, T. A. The evolving landscape of biomarkers for checkpoint inhibitor immunotherapy. *Nat. Rev. Cancer* **19**, 133–150 (2019). 30755690 10.1038/s41568-019-0116-x PMC6705396

[7] Agbor, A. A., Goksenin, A. Y., LeCompte, K. G., Hans, S. H. & Pursell, Z. F. Human Pol epsilon-dependent replication errors and the influence of mismatch repair on their correction. *DNA Repair (Amst.)* **12**, 954–963 (2013). 24051051 10.1016/j.dnarep.2013.08.012 PMC4520434

[8] Nicolas, E., Golemis, E. A. & Arora, S. POLD1: central mediator of DNA replication and repair, and implication in cancer and other pathologies. *Gene* **590**, 128–141 (2016). 27320729 10.1016/j.gene.2016.06.031 PMC4969162

[9] Palles, C. et al. Germline mutations affecting the proofreading domains of POLE and POLD1 predispose to colorectal adenomas and carcinomas. *Nat. Genet.* **45**, 136–144 (2013). 23263490 10.1038/ng.2503 PMC3785128

[10] Mertz, T. M., Baranovskiy, A. G., Wang, J., Tahirov, T. H. & Shcherbakova, P. V. Nucleotide selectivity defect and mutator phenotype conferred by a colon cancer-associated DNA polymerase delta mutation in human cells. *Oncogene* **36**, 4427–4433 (2017). 28368425 10.1038/onc.2017.22 PMC5542868

[11] Mertz, T. M., Sharma, S., Chabes, A. & Shcherbakova, P. V. Colon cancer-associated mutator DNA polymerase delta variant causes expansion of dNTP pools increasing its own infidelity. *Proc. Natl Acad. Sci. USA* **112**, E2467–E2476 (2015). 25827231 10.1073/pnas.1422934112 PMC4434702

[12] Goldsby, R. E. et al. High incidence of epithelial cancers in mice deficient for DNA polymerase delta proofreading. *Proc. Natl Acad. Sci. USA* **99**, 15560–15565 (2002). 12429860 10.1073/pnas.232340999 PMC137756

[13] Church, D. N. et al. DNA polymerase epsilon and delta exonuclease domain mutations in endometrial cancer. *Hum. Mol. Genet.* **22**, 2820–2828 (2013). 23528559 10.1093/hmg/ddt131 PMC3690967

[14] Wang, F. et al. Evaluation of POLE and POLD1 mutations as biomarkers for immunotherapy outcomes across multiple cancer types. *JAMA Oncol.* **5**, 1504–1506 (2019). 31415061 10.1001/jamaoncol.2019.2963 PMC6696731

[15] Chen, Y. X. et al. POLE/POLD1 mutation in non-exonuclease domain matters for predicting efficacy of immune-checkpoint-inhibitor therapy. *Clin. Transl. Med.* **11**, e524 (2021). 34586735 10.1002/ctm2.524 PMC8473642

[16] Rousseau, B. et al. PD-1 blockade in solid tumors with defects in polymerase epsilon. *Cancer Discov.* **12**, 1435–1448 (2022). 35398880 10.1158/2159-8290.CD-21-0521 PMC9167784

[17] Ma, X. et al. Functional landscapes of POLE and POLD1 mutations in checkpoint blockade-dependent antitumor immunity. *Nat. Genet.* **54**, 996–1012 (2022). 35817971 10.1038/s41588-022-01108-w PMC10181095

[18] Nguyen, B. et al. Genomic characterization of metastatic patterns from prospective clinical sequencing of 25,000 patients. *Cell* **185**, 563–575 e511 (2022). 35120664 10.1016/j.cell.2022.01.003 PMC9147702

[19] Ellrott, K. et al. Scalable open science approach for mutation calling of tumor exomes using multiple genomic pipelines. *Cell Syst.* **6**, 271–281 e277 (2018). 29596782 10.1016/j.cels.2018.03.002 PMC6075717

[20] Ayers, M. et al. IFN-gamma-related mRNA profile predicts clinical response to PD-1 blockade. *J. Clin. Invest.* **127**, 2930–2940 (2017). 28650338 10.1172/JCI91190 PMC5531419

[21] Cabrita, R. et al. Tertiary lymphoid structures improve immunotherapy and survival in melanoma. *Nature* **577**, 561–565 (2020). 31942071 10.1038/s41586-019-1914-8

[22] Jiang, P. et al. Signatures of T cell dysfunction and exclusion predict cancer immunotherapy response. *Nat. Med.* **24**, 1550–1558 (2018). 30127393 10.1038/s41591-018-0136-1 PMC6487502

[23] Danaher, P. et al. Pan-cancer adaptive immune resistance as defined by the Tumor Inflammation Signature (TIS): results from The Cancer Genome Atlas (TCGA). *J. Immunother. Cancer* **6**, 63 (2018). 29929551 10.1186/s40425-018-0367-1 PMC6013904

[24] Roelands, J. et al. An integrated tumor, immune and microbiome atlas of colon cancer. *Nat. Med.* **29**, 1273–1286 (2023). 37202560 10.1038/s41591-023-02324-5 PMC10202816

[25] Wang, F. et al. Genomic temporal heterogeneity of circulating tumour DNA in unresectable metastatic colorectal cancer under first-line treatment. *Gut* **71**, 1340–1349 (2022). 34489309 10.1136/gutjnl-2021-324852 PMC9185813

[26] Yuan, S. Q. et al. Residual circulating tumor DNA after adjuvant chemotherapy effectively predicts recurrence of stage II-III gastric cancer. *Cancer Commun. (Lond.)* **43**, 1312–1325 (2023). 37837629 10.1002/cac2.12494 PMC10693304

[27] Cohen, S. A., Liu, M. C. & Aleshin, A. Practical recommendations for using ctDNA in clinical decision making. *Nature* **619**, 259–268 (2023). 37438589 10.1038/s41586-023-06225-y

[28] Garmezy, B. et al. Clinical and molecular characterization of POLE mutations as predictive biomarkers of response to immune checkpoint inhibitors in advanced cancers. *JCO Precis. Oncol.* **6**, e2100267 (2022). 35108036 10.1200/PO.21.00267 PMC8820927

[29] Dong, S., Zakaria, H. & Hsiehchen, D. Non-exonuclease domain POLE mutations associated with immunotherapy benefit. *Oncologist* **27**, 159–162 (2022). 35274726 10.1093/oncolo/oyac017 PMC8914489

[30] Miao, D. et al. Genomic correlates of response to immune checkpoint therapies in clear cell renal cell carcinoma. *Science* **359**, 801–806 (2018). 29301960 10.1126/science.aan5951 PMC6035749

[31] Dai, J. et al. PBRM1 mutation as a predictive biomarker for immunotherapy in multiple cancers. *Front. Genet.* **13**, 1066347 (2022). 36699446 10.3389/fgene.2022.1066347 PMC9868445

[32] Reisman, D., Glaros, S. & Thompson, E. A. The SWI/SNF complex and cancer. *Oncogene* **28**, 1653–1668 (2009). 19234488 10.1038/onc.2009.4

[33] Mashtalir, N. et al. Modular organization and assembly of SWI/SNF family chromatin remodeling complexes. *Cell* **175**, 1272–1288 e1220 (2018). 30343899 10.1016/j.cell.2018.09.032 PMC6791824

[34] Hopson, S. & Thompson, M. J. BAF180: its roles in DNA repair and consequences in cancer. *ACS Chem. Biol.* **12**, 2482–2490 (2017). 28921948 10.1021/acschembio.7b00541

[35] Yang, Q. et al. Comprehensive analyses of PBRM1 in multiple cancer types and its association with clinical response to immunotherapy and immune infiltrates. *Ann. Transl. Med.* **9**, 465 (2021). 33850862 10.21037/atm-21-289 PMC8039713

[36] Pan, D. et al. A major chromatin regulator determines resistance of tumor cells to T cell-mediated killing. *Science* **359**, 770–775 (2018). 29301958 10.1126/science.aao1710 PMC5953516

[37] Chen, S., Zhou, Y., Chen, Y. & Gu, J. fastp: an ultra-fast all-in-one FASTQ preprocessor. *Bioinformatics* **34**, i884–i890 (2018). 30423086 10.1093/bioinformatics/bty560 PMC6129281

[38] Li, H. & Durbin, R. Fast and accurate long-read alignment with Burrows-Wheeler transform. *Bioinformatics* **26**, 589–595 (2010). 20080505 10.1093/bioinformatics/btp698 PMC2828108

[39] Chen, S. et al. Gencore: an efficient tool to generate consensus reads for error suppressing and duplicate removing of NGS data. *BMC Bioinforma.* **20**, 606 (2019). 10.1186/s12859-019-3280-9 PMC6933617 31881822

[40] Li, H. et al. The Sequence Alignment/Map format and SAMtools. *Bioinformatics* **25**, 2078–2079 (2009). 19505943 10.1093/bioinformatics/btp352 PMC2723002

[41] Koboldt, D. C. et al. VarScan 2: somatic mutation and copy number alteration discovery in cancer by exome sequencing. *Genome Res.* **22**, 568–576 (2012). 22300766 10.1101/gr.129684.111 PMC3290792

[42] Wang, K., Li, M. & Hakonarson, H. ANNOVAR: functional annotation of genetic variants from high-throughput sequencing data. *Nucleic Acids Res.* **38**, e164 (2010). 20601685 10.1093/nar/gkq603 PMC2938201

[43] Chen, T. et al. The genome sequence archive family: toward explosive data growth and diverse data types. *Genomics Proteom. Bioinforma.* **19**, 578–583 (2021). 10.1016/j.gpb.2021.08.001 PMC9039563 34400360

[44] CNCB-NGDC Members and Partners. Database Resources of the National Genomics Data Center, China National Center for Bioinformation in 2022. Nucleic Acids Res. 50, D27–D38 (2022). 10.1093/nar/gkab951 PMC8728233 34718731

