# Supplementary material for: A phase II clinical trial of toripalimab in advanced solid tumors with polymerase epsilon/polymerase delta (POLE/POLD1) mutation
Source: Signal Transduct Target Ther. 2024 Sep 2;9:227. doi: 10.1038/s41392-024-01939-5 (PMC11366758; doi:10.1038/s41392-024-01939-5)
Supplement: Supplementary file 1 — Study Protocol [file 41392_2024_1939_MOESM1_ESM.docx]

**A Phase 2 Clinical Study of Toripalimab in Patients with POLE or POLD1 Mutated and Non-MSI-H Advanced Solid Tumors**

**Principal Investigator: Prof. Ruihua Xu and Feng Wang**

**Site Location: Sun Yat-sen University Cancer Center**

**PROTOCOL REVISION HISTORY**

| **Summary of Changes** |
| --- |
| **2.3 Exploratory Endpoints**  Blood samples, pathological slides, saliva and stool samples will be collected for exon sequencing and detection of inflammatory factors, metabolites and bacteria to further explore the factors affecting the efficacy of immunotherapy for participants with POLE or POLD1 mutated and MSS or MSI-L advanced solid tumors.  3.3.4 Exploratory Analysis  To describe the detection rate of ctDNA and its correlation with immunotherapy, and the consistency of ctDNA with tissue detection, and the dynamic variation of ctDNA in immunotherapy efficacy assessment, and the composition of intestinal microorganism pre- and post-treatment, the relationship between intestinal flora alpha diversity and beta diversity and immunotherapy response as well as between intestinal microorganism alpha diversity and beta diversity and treatment toxicity and tolerance.  **3.4 Sample collection**  Collection of blood samples: Blood samples will be collected from eligible patients at baseline and subsequent imaging evaluation (every 6 weeks in the first year, and every 12 weeks thereafter) after enrollment, regardless of discontinuation. Plasma and white blood cells will be separated immediately, and stored in a -80°C refrigerator.  Collection of fecal samples: Fecal samples will be collected from eligible patients at baseline and subsequent imaging evaluation (every 6 weeks in the first year, and every 12 weeks thereafter) after enrollment, regardless of discontinuation. Fecal samples will be stored in a -80°C refrigerator.  Collection of saliva samples: Saliva samples will be collected from eligible patients at baseline and subsequent imaging evaluation (every 6 weeks in the first year, and every 12 weeks thereafter) after enrollment, regardless of discontinuation. Saliva samples will be stored in a -80°C refrigerator after aliquoting.  Collection of tissue samples: Formalin-fixed and paraffin-embedded tissue samples will be collected from eligible patients after enrollment.  **4.3 Discontinuation criteria**   - Intolerable toxicity; - PD (with exception of those who were evaluated by the Investigator and might be benefit from further Toripalimab treatment); - Need to receive other anti-tumor treatment or any concomitant treatment which has a significant effect on the safety and efficacy of the investigational product during the study; - Termination of the study by Sun Yat-Sen University Cancer Center   **4.4 Withdrawal criteria**  Reasons for discontinuation of study treatment or withdrawal from the study may include, but are not limited to:   - Patients withdraw the ICF and request to withdraw from the study - Any medical condition that the Investigator determines may jeopardize the patient’s safety if he or she continues in the study or continues treatment with study drug - The Investigator determines it is in the best interest of the patient - Patient noncompliance (eg, not complying with protocol required visits, and assessments) - Pregnancy.   Patient who are withdrawn prematurely from the study will be asked to complete study assessments.  **7.1 Management of Special Adverse Events**  **7.1.1 Infusion Reactions**  Clinical symptoms of infusion reactions include fever, chills, nausea, pruritus, vasogenic edema, hypotension, headache, tracheospasm, urticaria, skin rashes, vomiting, muscle pain, and somnolence or hypertension. Possible serious reactions include acute respiratory distress syndrome, myocardial infarction, ventricular fibrillation and cardiogenic shock. Therefore, the subjects in this study must be closely observed for relevant clinical symptoms. ECG monitoring and rescue medication (including but not limited to epinephrine, glucocorticoids, antihistamines, bronchodilators, and oxygen) are required for rescue if a serious reaction occurs. If an infusion reaction or suspected event of CTCAE Grade 2 or higher occurs, the infusion of Toripalimab must be stopped immediately. Subjects will be informed of possible delayed post-infusion symptoms and asked to contact their study doctor if they experience these symptoms. The subjects must be treated according to the local best practice if a serious allergic reaction occurs.  **7.1.2 Serious Allergic Reactions**  Antibody administration may cause allergic reactions. Appropriate drugs and medical equipment must be provided immediately to treat acute allergic reactions, and the study personnel must be trained to identify and treat allergic reactions. The study site must be equipped with first-aid team and equipment, and have the ability to admit the subject into an intensive care unit if necessary. The subject must be given emergent treatment according to local medical practices when a serious allergic reaction occurs. When a subject experiences such symptoms, he (she) must inform the Investigator immediately. In the guidelines of National Institute of Allergy and Infectious Diseases (NIAID) and Food Allergy & Anaphylaxis Network (FAAN), anaphylaxis is defined as severe allergic reactions with rapid onset and possible death. These three types of allergic reactions, as shown below, cover 80% (category 1) to 95% of the cases (all three categories).   1. Acute onset (minutes to hours) of allergic reaction involving the skin, mucosal tissues, or both, (e.g., generalized urticaria, itching or flushing, swelling lips and tongue) and at least one of the following: 2. Dyspnea (e.g., dyspnea, stridor-bronchospasm, stridor, peak expiratory flow (PEF) decreased, hypoxemia); 3. Reduced blood pressure (BP) or associated symptoms of end-organ dysfunction (e.g., hypotensive shock, syncope, incontinence). 4. The subject may experience two or more of the following after exposure to an allergen (minutes to hours) : 5. Involvement of the skin-mucosal tissue (e.g., generalized hives, pruritus, swollen lips and tongue); 6. Dyspnea (e.g., dyspnea, stridor-bronchospasm, stridor, PEF decreased, hypoxemia); 7. Reduced blood pressure or associated symptoms (e.g., hypotensive shock, syncope, incontinence); 8. Persistent gastrointestinal symptoms (e.g., abdominal pain, vomiting). 9. Blood pressure decreases in patients exposed to known allergens (minutes to hours): 10. Infants and children: low systolic blood pressure (age-specific) or less than 30% reduction in systolic blood pressure; 11. Adults: Systolic blood pressure less than 90 mm Hg or more than 30% lower than the baseline. |

**1. Study Background**

Immunotherapy, as a novel therapy for malignant tumors, has made great progress in recent years. The 2018 Nobel Prize in Physiology or Medicine was awarded to American immunologist James P Alison and Japanese immunologist TasukuHonjo for their contributions to the tumor immunology field. Studies have shown that tumor cells can inhibit the activity of T cells by expressing ligands of immune checkpoint pathways, thus escaping from the killing effect of the immune system. Therefore, blocking immune checkpoint pathways can enhance T cells proliferation, survival, and killing activity and achieve tumor inhibition. Compared with traditional chemical and targeted therapy, immune checkpoint antibody drugs have the following advantages: a). more broad-spectrum anti-tumor effect; b). enhanced specific killing effect on tumor cells and reduced toxicity; c). ability to enter the targeted cancer tissue; d). ability to adjust the immune response to new tumor antigens to achieve the immune response to both the original lesion and metastasis; e). ability to sustainably inhibit tumor cells. Food and Drug Administration (FDA) approved the first cellular immunotherapy for prostate cancer in 2010, and then in 2011 approved Ipilimumab (trade name: Yervoy), a monoclonal antibody against cytotoxic T lymphocyte-associated antigen-4 (CTLA-4 ) developed by Bristol-Myers Squibb and the first immune checkpoint antagonist marketed worldwide. In 2014, Nivolumab (trade name: Opdivo), a programmed cell death protein 1 (PD-1) inhibitor jointly developed by Ono Pharmaceutical and Bristol-Myers Squibb, and Pembrolizumab (trade name: Keytruda), a PD-1 inhibitor developed by Merck Sharp & Dohme Corp., were also approved for marketing. Currently, as one of the regimens for the treatment of tumors, immunotherapy mainly includes cytokine therapy, therapeutic vaccine, adoptive T-cell therapy (ACT) and immune checkpoint blocking or immune co-stimulatory pathway activators, including CTLA-4/PD-1/PD-L1 (programmed cell death ligand 1) antibody drugs. In recent years, clinical studies have demonstrated that checkpoint-specific targeted monoclonal antibody drugs have shown good efficacy alone or in combination with chemotherapy, thus making immunotherapy more critical in treating a variety of tumors.

PD-1 is an important inhibitory receptor expressed mainly on the surface of T cells, B cells, monocytes, and NK cells [[1](#references1)]. The ligands for PD-1 are the B7 homologous proteins PD-L1 (also known as B7-H1) and PD-L2 (also known as B7-DC), where PD-L1 is widely expressed in various tumor and immune cells. In the intracellular domain of PD-1, the immunoreceptor tyrosine-based switch motif (ITSM) mediates the recruitment of SHP family phosphatases and the inhibition of T cell activation signals. PD-1/PD-L1 binding plays a vital role in down-regulating T cell activation and maintaining peripheral immune tolerance, while tumor cells can inhibit T cell activation by expressing PD-L1 and then interacting with PD-1, thus escaping from the killing effect of the immune system.

Currently, the clinical trials in PD-1 targeted agents at home and overseas have been conducted on advanced melanoma, non-small cell lung cancer, renal cell carcinoma, gastric cancer, ovarian cancer, triple-negative breast cancer, colorectal cancer, pancreatic adenocarcinoma, hepatocellular carcinoma, prostate cancer, transitional cell carcinoma, head and neck squamous cell carcinoma, head and neck cancer, adult acute myeloid leukemia, chronic granulocytic leukemia, multiple myeloma, myelodysplastic syndrome, cervical carcinoma and glioblastoma. Completed clinical trials and the interim results of partial trials revealed that the PD-1 antibody is significantly superior to the previous therapeutic approaches showing better efficacy and higher safety.

Toripalimab, developed by Shanghai Junshi Biosciences Co., Ltd., is a neutralizing blocking antibody targeting on human PD-1. It binds to PD-1 with high affinity and selectively blocks the binding of PD-1 with its ligand PD-L1 and PD-L2 to activate T lymphocytes, enhance the proliferation of lymphocytes and secretion of cytokines, especially IFN-γ.

The preclinical pharmacodynamic trial has proved that, in animal models with graft-versus-host disease (GVHD) induced by adoptive transfer of human PBMC, Toripalimab could significantly stimulate the proliferation of CD4+ and CD8+ T cells to promote the activation of human effector/memory T cells. Meanwhile, the study in animal models implanted with 624 MEL human melanomas has proved that, Toripalimab in combination with cytotoxic T lymphocytes (CTL) could eliminate the immunosuppressive effects and enhance the killing effect of CTL on tumor cells, thus achieving the expected good therapeutic effect. The difference in CDR sequences and structure of the antigen-binding site between Toripalimab, Nivolumab and Pembrolizumab mainly manifests in 6 CDR sequences, which determines the various physical, chemical and biological characteristics of the three drugs. Based on the affinity test results using SPR or ELISA methods, Toripalimab has a higher affinity than Pembrolizumab and Nivolumab.

There are 3 ongoing Phase 1a studies of Toripalimab (CTR20160274, CTR20160187 and CTR20160176) in China. The first-in-human study (Study No.: JS001-I-CRP-1.3) conducted at Sun Yat-sen University Cancer Center is a dose-escalation study, with the dose escalation following the 3+3 principle. Patients are treated at 0.3 mg/kg, 1 mg/kg, 3 mg/kg and 10 mg/kg, respectively, once every 2 weeks. The current first-in-human study data of Toripalimab showed that no dose-limiting toxicity (DLT) has been observed, and the available safety data and pharmacokinetic data demonstrated acceptable safety profile of Toripalimab in patients. The study showed preliminary efficacy of Toripalimab in patients with gastric, esophageal, nasopharyngeal, and head and neck cancer [[2](#references2)].

Mismatch repair (MMR) is a well-established biomarker in predicting the response to anti-PD-1 therapy [[3](#references3),[4](#references4)]. Previous clinical studies have shown that anti-PD-1 therapy has an objective response rate of 53% for solid tumors with deficient mismatch repair (dMMR)[[4](#references4)]. In addition, the predictive effect of tumor mutational load on the response to anti-PD-1 therapy has been increasingly appreciated. In 2015, a study showed that PD-1/PD-L1 antibodies were significantly more effective in lung cancer patients with high tumor mutational load than with low tumor mutational load [[5](#references5)]. In addition, high tumor mutational load also contributes to predicting the response to tumor immunotherapy in bladder cancer and melanoma [[6](#references6),[7](#references7)]. In 2017, a study based on tumor type showed that the tumor type with a higher mutational load was associated with a higher objective response rate with anti-PD-1 therapy [[8](#references8)]. Preliminary studies by our team have shown that POLE mutation may lead to an increased mutational load in the tumor type with proficient mismatch repair (pMMR). The efficacy of anti-PD-1 therapy in malignant gliomas and endometrial cancer with POLE mutation has been reported in individual cases [[9](#references9),[10](#references10)].

The purpose of this study is to preliminarily evaluate the safety and efficacy of Toripalimab in participants with POLE or POLD1 mutated and non-MSI-H advanced solid tumors to support further large-scale clinical studies.

**2. Study Objectives**

**2.1 Primary Objective:**

To investigate the overall response rate (proportion of patients who had a best overall response of either complete response [CR] or partial response [PR] according to RECIST 1.1) of Toripalimab in participants with POLE/POLD1 mutated and MSS/MSI-L advanced solid tumors.

**2.2 Secondary Objectives:**

- To investigate the disease control rate (defined as the proportion of patients with the best overall response of CR, PR, or stable disease [SD]) of Toripalimab in participants with POLE or POLD1 mutated and MSS or MSI-L advanced solid tumors.
- To investigate the overall survival (OS) of Toripalimab in participants with POLE or POLD1 mutated and MSS or MSI-L advanced solid tumors.
- To investigate the progress free survival (PFS) of Toripalimab in participants with POLE or POLD1 mutated and MSS or MSI-L advanced solid tumors.
- To evaluate the safety of Toripalimab in participants with POLE or POLD1 mutated and MSS or MSI-L advanced solid tumors.

**2.3 Exploratory Endpoints**

Blood samples, pathological slides, saliva and stool samples will be collected for exon sequencing and detection of inflammatory factors, metabolites and bacteria to further explore the factors affecting the efficacy of immunotherapy for participants with POLE or POLD1 mutated and MSS or MSI-L advanced solid tumors.

**3. Study Design**

**3.1 Overall Design of Clinical Trial**

This is an open-label, single-arm, phase 2 clinical study to investigate the efficacy and safety of Toripalimab in participants with POLE or POLD1 mutated and MSS or MSI-L advanced solid tumors. The study drug, Toripalimab, will be given at 240 mg q3w in 3-week dosing cycles.

**3.2 Sample size**

Simon's two-stage optimal design method will be used to calculate the sample size. Assuming P1 = 0.3 (best), P0 = 0.1 (worst), α = 0.05, β = 0.1, eighteen patients will be enrolled in the first stage, and the trial will be terminated if only 2 or fewer patients are eligible. Otherwise, the second stage will be started, and an additional 17 patients will be enrolled in the stage, i.e., the total number of patients enrolled is 35. There will be a 90% probability to confirm the assumed differences in the primary endpoint of the study if more than 6 are available in both stages.

**3.3 Statistical Analysis**

Computer-based SPSS or R software package will be used for statistical analyses. In general, the continuous variables will be statistically described by the number of patients, mean, median, standard deviation, minimum and maximum; the categorical variables and grade variables will be statistically described by the frequency and percentage of each category or grade; unless otherwise specified, no missing values will be included in the calculation of percentage.Unless otherwise specified, all statistical tests will be performed using a two-tailed test (α = 0.05) and the two-sided 95% confidence interval (CI) will be calculated. The Kaplan-Meier method and log-rank method will be used for survival analysis.

3.3.1 Data analysis sets

Full Analysis Set (FAS): All subjects who are eligible after screening and assigned with the enrollment numbers and have received at least one dose of study drug will be included in the full analysis set, which will be used to analyze patient demographics and baseline characteristics.

Efficacy Analysis Set: Efficacy analysis will be performed for all patients who have baseline radiological tumor assessment, received at least one dose of study drug per protocol and have at least one post-baseline tumor assessment.

Safety Analysis Set (SAS): All the patients who have received at least one dose of the investigational product after enrollment and have safety records after administration will be included in the safety analysis set. This dataset will be used for safety analyses.

3.3.2 Demographics and Other Baseline Characteristics

The demographics and other baseline characteristics will be summarized in a table and descriptively analyzed by descriptive statistics.

3.3.3 Safety Analysis

For safety analysis, toxicity will be evaluated as per NCI-CTCAE criteria version 5.0. The changes in vital signs, physical examination, 12-lead ECG, laboratory tests, imaging examination before and after treatment administration will be summarized by descriptive statistics. All adverse events will be coded with MedDRA. The treatment-related adverse events and serious adverse events will be classified, summarized and analyzed by the organ system class and preferred term, and the number and percentage of subjects experiencing adverse events will be calculated.

3.1.5 Efficacy Analysis

The number and percentage of patients who have achieved complete response (CR), partial response (PR), stable disease (SD) and progressive disease (PD) will be summarized by descriptive statistics, respectively, and the objective response rate and disease control rate and their 95% confidence intervals will be calculated. Progression free survival (PFS) is defined as time from the first dose to first documentation of disease progression or death from any cause (whichever occurs first). Subjects without any event during follow-up or study treatment will be censored at the last tumor assessment. Subjects without post-baseline assessment will be censored at the date of enrollment. Overall survival (OS) is defined as time from the first dose to death from any cause. Subjects without any event will be censored at the last date known to be alive. Subjects without any follow-up information will be censored at the date of enrollment.

3.3.4 Exploratory Analysis

To describe the detection rate of ctDNA and its correlation with immunotherapy, and the consistency of ctDNA with tissue detection, and the dynamic variation of ctDNA in immunotherapy efficacy assessment, and the composition of intestinal microorganism pre- and post-treatment, the relationship between intestinal flora alpha diversity and beta diversity and immunotherapy response as well as between intestinal microorganism alpha diversity and beta diversity and treatment toxicity and tolerance.

**3.4 Sample collection**

Collection of blood samples: Blood samples will be collected from eligible patients at baseline and subsequent imaging evaluation (every 6 weeks in the first year, and every 12 weeks thereafter) after enrollment, regardless of discontinuation. Plasma and white blood cells will be separated immediately, and stored in a -80°C refrigerator.

Collection of fecal samples: Fecal samples will be collected from eligible patients at baseline and subsequent imaging evaluation (every 6 weeks in the first year, and every 12 weeks thereafter) after enrollment, regardless of discontinuation. Fecal samples will be stored in a -80°C refrigerator.

Collection of saliva samples: Saliva samples will be collected from eligible patients at baseline and subsequent imaging evaluation (every 6 weeks in the first year, and every 12 weeks thereafter) after enrollment, regardless of discontinuation. Saliva samples will be stored in a -80°C refrigerator after aliquoting.

Collection of tissue samples: Formalin-fixed and paraffin-embedded tissue samples will be collected from eligible patients after enrollment.

**4. Eligibility Criteria**

**4.1 Inclusion Criteria**

- 18-75 years old
- Patients with pathologically confirmed advanced solid tumors with POLE/POLD mutation
- Refractory or intolerant to systemic chemotherapy or target therapy, or refuse any chemotherapy or targeted therapy
- Germline or somatic mutations in POLE and POLD (synonymous mutations is excluded)
- No history of radiotherapy or received non-targeted radiotherapy outside the target lesions for this study more than 4 weeks ago before the first dose of study treatment
- MSS (microsatellite sability) or MSI-L (microsatellite instability-low) or pMMR status
- Participants must have at least one measurable site of disease according to Response Evaluation Criteria in Solid Tumors (per RECIST version 1.0) criteria. Lesions previously treated with radiotherapy should not be regarded as target lesions unless there is a definite progression of the lesion after radiotherapy.
- ECOG performance status 0-2
- Estimated life expectancy ≥ 3 months
- Aspartate aminotransferase (AST) and alanine aminotransferase (ALT) ≤ 2.5 x (5 x in participants with liver metastasis) upper limit of normal (ULN)
- Albumin ≥3.0 g/dL
- Alkaline phosphatase ≤ 2.5 x ULN
- Serum bilirubin <1.5 mg/dL
- Creatinine ≤ ULN.
- Absolute neutrophil count (ANC) ≥ 1500/mm^3^, hemoglobin (Hb) > 9g/dl, and platelets > 100,000/mm^3^
- Having signed a written informed consent form

**4.2 Exclusion Criteria**

- Patients with confirmed or suspected brain metastases or carcinomatous meningitis
- Microsatellite instability-high (MSI-H) or deficient DNA mismatch repair (dMMR)
- Prior treatment with PD-1 inhibitors, PD-L1 inhibitors, PD-L2 inhibitors, or CTLA-4 inhibitors (or other inhibitors in T cell co-stimulatory signals or checkpoint pathways)
- Known history or evidence of cytotoxic drug therapy, biologic drug therapy (such as monoclonal antibodies), immunotherapy (such as interleukin 2 or interferon), or other investigational drugs therapy within 4 weeks prior to first dose of study drug
- Having any active autoimmune diseases or history of autoimmune diseases (including but not limited to interstitial pneumonia, uveitis, enteritis, hepatitis, hypophysitis, nephritis, hyperthyroidism, hypothyroidism; subjects with vitiligo or who had a complete response of asthma in childhood and do not require any intervention after adulthood can be included; asthma requiring medical intervention with a bronchodilator cannot be included)
- Patients with active tuberculosis. Known history of antituberculosis drugs treatment in 1 year before the first dose of study treatment
- Having comorbidities requiring chronic therapy with immunosuppressive drugs, or systemic or topical corticosteroids at immunosuppressive doses (> 10 mg/day of prednisone or other equivalent steroids)
- Administration of an anti-infection vaccine (e.g. influenza vaccine, chickenpox vaccine) within 4 weeks prior to enrollment
- Having heart failure, coronary heart disease and myocardial infarction within 6 months prior to enrollment
- Prisoners or patients who are involuntarily incarcerated.
- Other prior malignancy active within the previous 5 years except for non-melanoma skin cancer
- Known or suspected allergy to the study drug or allergic to any study related drug administered
- Known history of testing positive for human immunodeficiency virus (HIV) or known acquired immunodeficiency syndrome (AIDS).
- Positive test for HbsAg and HBV-DNA copy numbers (≥ 1000cps/ml)
- Positive blood screening for chronic hepatitis C (positive HCV antibody)
- Any medical disorder or condition that, in the opinion of the Investigator, may affect the compliance or the signing of informed consent, or is inappropriate for participation in this clinical study
- Woman of child bearing potential who are pregnant or breastfeeding, or women with a positive pregnancy test at enrollment or prior to administration of study medication.

**4.3 Discontinuation criteria**

- Intolerable toxicity;
- PD (with exception of those who were evaluated by the Investigator and might be benefit from further Toripalimab treatment);
- Need to receive other anti-tumor treatment or any concomitant treatment which has a significant effect on the safety and efficacy of the investigational product during the study;
- Termination of the study by Sun Yat-Sen University Cancer Center

**4.4 Withdrawal criteria**

Reasons for discontinuation of study treatment or withdrawal from the study may include, but are not limited to:

- Patients withdraw the ICF and request to withdraw from the study
- Any medical condition that the Investigator determines may jeopardize the patient’s safety if he or she continues in the study or continues treatment with study drug
- The Investigator determines it is in the best interest of the patient
- Patient noncompliance (eg, not complying with protocol required visits, and assessments)
- Pregnancy.

Patient who are withdrawn prematurely from the study will be asked to complete study assessments.

**4.5 Elimination Criteria**

Before statistical analysis of data, the statistician and Principal Investigator will discuss and determine whether any individual cases require exclusion. In case of any one of the following situations, the statistician and the Investigator should comprehensively judge whether the subject’s data should be removed based on factors such as the degree of subject completion of the study and the reason for withdrawal, and exclude the subject in the per-protocol set with the relevant explanations attached:

- Subjects who fail to meet the inclusion criteria or meet the exclusion criteria after participating in the study are determined as major protocol violators;
- Subjects who fail to comply with the study plan during the study (poor compliance, such as patients who have not used investigational product, who are unable to receive the evaluation of efficacy and safety as required in the study protocol or who have no data);
- Subjects who have other major protocol deviations or protocol violations during the study have a serious impact on drug efficacy and safety in the judgment of the Investigator and statistician, except for the above situations

1. **Treatment Procedure and Treatment after Progression of Disease**

The Investigator will explain all study procedures to each subject and obtain the informed consent form signed voluntarily by the subject. The subjects can enter study related assessment and procedure only after signing the informed consent form. Subjects’ demographic data will be obtained, including date of birth, gender, nationality, etc. For female subjects, whether there is childbearing potential should be recorded. Medical history and treatment history of colorectal cancer will be recorded, including diagnosis, date of diagnosis, previous treatment regimens (chemotherapy, targeted therapy, radiotherapy etc.) and start/stop dates, best treatment assessment, date of disease progression. Other past medical history, surgical history, allergy history; drugs used within 4 weeks before signing informed consent form will be recorded, including prescription drugs, over-the-counter drugs and Chinese medicine.

Complete physical examination and examination of vital signs(including body temperature, respiration, blood pressure and pulse) will be performed during screening period. Examination of vital signs. Complete physical examination will include measurements of whole-body organ systems (general body surface, skin, head and neck, chest [heart, lung, breast], abdomen [gastrointestinal tract, liver, gallbladder], back, urinary system, limbs, nervous system, lymph nodes, etc.), height and body weight.

Cardiac ultrasound examination, electrocardiogram, hematology, blood biochemistry, urinalysis, stool routine, thyroid function tests, and coagulation will be performed in screening period. Hematology includes complete blood count and differential count. Blood biochemistry includes aspartate aminotransferase (AST), alanine aminotransferase (ALT), alkaline phosphatase (ALP), γ-glutamyl transpeptidase (γ-GT), total bilirubin (TBIL), direct bilirubin (DBIL), total protein (TP), albumin (ALB), blood urea nitrogen (BUN)/urea (Urea), amylase or lipase, creatinine (Cr), phosphate (PO33-), sodium (Na+), potassium (K+), chloride (Cl-), calcium (Ca2+), glucose (GLU) and glycosylated hemoglobin (HbA1c). Urinalysis includes pH value, glucose, urine protein, white blood cell and red blood cell. Coagulation test includes prothrombin time (PT), thrombin time (TT), activated partial thromboplastin time (APTT) and international normalized ratio (INR). Thyroid function test includes thyroid stimulating hormone (TSH), free thyroid hormone (T4) and free triiodothyroxine (T3). Virological tests will be performed in screening period. Virological tests include hepatitis B surface antigen (HbsAg), hepatitis B surface antibody (HbsAb), hepatitis B e antigen (HbeAg), hepatitis B e antibody (HbeAb), hepatitis B core antibody (HbcAb), hepatitis B virus DNA (HBV-DNA), human immunodeficiency virus (HIV Ab) and hepatitis C virus antibody (HCV Ab). To be enrolled, pregnancy test result of female subjects must be negative. Radiological examination of tumor will be performed in screening period. Chest, abdominal, pelvic CT or MRI scan has to be performed, and brain CT/MRI scan and bone scan will be performed if clinically indicated. The subject should have corresponding CT or MRI examination results obtained within 4 weeks prior to initiation of study treatment.

After enrollment, patients will receive 2 years of treatment by intravenous infusion of Toripalimab 240 mg q3w on Day 1 of each cycle. The treatment will be discontinued if the patient experiences intolerable toxicity and disagrees with continuing in the clinical study, or PD or death occurs.

After PD, if the Investigator evaluates that they may continue to benefit from Toripalimab, the subject may continue the investigational product. If the subject experiences PD again after continuous dosing of the investigational product, he/she should withdraw from the study permanently. The Investigator should determine re-progression based on RECIST version 1.1, taking the result of tumor evaluation at the time of the first progression as the baseline value.

Subjects cannot continue to receive the investigational product after PD unless the following criteria are met:

- The benefit of a continued dose of Toripalimab overweighs the risk as judged by the Investigator;
- ECOG performance status ≤ 2.

**6. Follow-up**

**6.1 Treatment Period Visits**

- Physical examination;
- Vital signs;
- ECOG performance status;
- Laboratory tests: hematology, blood biochemistry, urinalysis, stool routine, coagulation, thyroid function. laboratory tests during the treatment period should be completed before each dose, except for C1D1. Blood samples should not be collected earlier than 7 days before each dose. The dose can only be started after the laboratory test results meet the criteria as determined by the Investigator;
- 12-lead ECG;
- Thyroid function: once at baseline and every two cycles subsequently. In case of any clinically significant changes in thyroid function, endocrinology consultation is recommended to rule out pituitary function impairments;
- Tumor evaluation: Baseline and subsequent imaging should be conducted by the same imaging method (CT or MRI) and evaluated by the same reader, if possible. Cycles should be calculated from C1D1, and the tumor evaluation should be performed once every 6 weeks in the first year and every 12 weeks thereafter, regardless of discontinuation. Subjects suspected of disease progression prior to the next scheduled tumor evaluation should receive an unscheduled tumor evaluation;
- Adverse events.

In this study, the recruitment and follow-up periods are both 12 months. The enrollment of all patients and the observation of various endpoints will be completed during the period.

**Safety monitoring period:** The safety monitoring will be performed after signing the informed consent form and will continue until 30 days after the last dose of the study drug.

**Observation period for efficacy endpoints:** participants will be observed until PFS is determined for all patients. The specific dates of the following endpoint events, whichever occurs first, shall be recorded to determine the PFS of the patient:Objective tumor progression of disease (PD), Death, Receipt of other anti-tumor therapy after enrollment and treatment.

**6.2 End of Treatment Visit**

- Physical examination;
- Vital signs;
- ECOG performance status;
- Laboratory tests: hematology, blood biochemistry, urinalysis, stool routine, coagulation, thyroid function.;
- 12-lead ECG;
- Thyroid function;
- Tumor evaluation:
- Baseline and subsequent imaging should be conducted by the same imaging method (CT or MRI) and evaluated by the same reader, if possible.
- Concomitant medication/concomitant therapy;
- Collection of adverse events/serious adverse events.

**6.3 Survival Follow-up**

Adverse events should be recorded until 60 days after the last dose of the investigational product or until the start of any other anticancer therapy, whichever occurs first. After the period, only ongoing serious adverse events considered by the Investigator to be related to study treatment will be recorded. For unresolved adverse events or abnormal laboratory test results considered related to study treatment, the subject will continue to be followed until the events are resolved or return to baseline. Subjects who end the treatment for reasons other than PD should be followed for tumor evaluation every 6 weeks in the first year and every 12 weeks thereafter until PD, death, start of new anti-tumor therapy, or withdrawal of consent. Thereafter, the information on the subsequent anti-tumor treatment and survival status of subjects will be collected every 3 months. Baseline and subsequent imaging should be conducted by the same imaging method (CT or MRI) and evaluated by the same reader, if possible. Tumor evaluation should be performed according to the original imaging schedule. Subjects suspected of disease progression prior to the next scheduled tumor evaluation should receive an unscheduled tumor evaluation. For subjects with PD, the information on subsequent anti-tumor treatment and survival status will be collected every 3 months from the end of treatment visit.

**7. Management of Adverse Events**

Toripalimab may be associated with the following potentially significant immune-related adverse events: immune-related hepatitis, pneumonitis, colitis, pancreatitis, endocrine disorders (hypothyroidism, hyperthyroidism, adrenal cortex insufficiency). In addition, immune-related adverse reactions also include the following clinically significant events: exfoliative dermatitis, uveitis, arthritis, myocarditis, pancreatitis, hemolytic anemia, partial seizures (which may occur in subjects with inflammation of the cerebral hemispheres), adrenal insufficiency, myasthenia gravis, optic neuritis, and rhabdomyolysis. Suspected immune-related adverse reactions should be closely observed for relevant system functions, and an adequate evaluation should be warranted to determine the etiology and rule out other causes. Overall, Toripalimab should be suspended or discontinued and/or symptomatic treatment, such as corticosteroids, should be administered depending on the severity of the event. The subjects will be treated according to the treatment principles for immunotherapy adverse reactions provided by the Chinese Society Of Clinical Oncology (CSCO).

**7.1 Management of Special Adverse Events**

**7.1.1 Infusion Reactions**

Clinical symptoms of infusion reactions include fever, chills, nausea, pruritus, vasogenic edema, hypotension, headache, tracheospasm, urticaria, skin rashes, vomiting, muscle pain, and somnolence or hypertension. Possible serious reactions include acute respiratory distress syndrome, myocardial infarction, ventricular fibrillation and cardiogenic shock. Therefore, the subjects in this study must be closely observed for relevant clinical symptoms. ECG monitoring and rescue medication (including but not limited to epinephrine, glucocorticoids, antihistamines, bronchodilators, and oxygen) are required for rescue if a serious reaction occurs. If an infusion reaction or suspected event of CTCAE Grade 2 or higher occurs, the infusion of Toripalimab must be stopped immediately. Subjects will be informed of possible delayed post-infusion symptoms and asked to contact their study doctor if they experience these symptoms. The subjects must be treated according to the local best practice if a serious allergic reaction occurs.

**7.1.2 Serious Allergic Reactions**

Antibody administration may cause allergic reactions. Appropriate drugs and medical equipment must be provided immediately to treat acute allergic reactions, and the study personnel must be trained to identify and treat allergic reactions. The study site must be equipped with first-aid team and equipment, and have the ability to admit the subject into an intensive care unit if necessary. The subject must be given emergent treatment according to local medical practices when a serious allergic reaction occurs. When a subject experiences such symptoms, he (she) must inform the Investigator immediately. In the guidelines of National Institute of Allergy and Infectious Diseases (NIAID) and Food Allergy & Anaphylaxis Network (FAAN), anaphylaxis is defined as severe allergic reactions with rapid onset and possible death. These three types of allergic reactions, as shown below, cover 80% (category 1) to 95% of the cases (all three categories).

1. Acute onset (minutes to hours) of allergic reaction involving the skin, mucosal tissues, or both, (e.g., generalized urticaria, itching or flushing, swelling lips and tongue) and at least one of the following:
2. Dyspnea (e.g., dyspnea, stridor-bronchospasm, stridor, peak expiratory flow (PEF) decreased, hypoxemia);
3. Reduced blood pressure (BP) or associated symptoms of end-organ dysfunction (e.g., hypotensive shock, syncope, incontinence).
4. The subject may experience two or more of the following after exposure to an allergen (minutes to hours) :
5. Involvement of the skin-mucosal tissue (e.g., generalized hives, pruritus, swollen lips and tongue);
6. Dyspnea (e.g., dyspnea, stridor-bronchospasm, stridor, PEF decreased, hypoxemia);
7. Reduced blood pressure or associated symptoms (e.g., hypotensive shock, syncope, incontinence);
8. Persistent gastrointestinal symptoms (e.g., abdominal pain, vomiting).
9. Blood pressure decreases in patients exposed to known allergens (minutes to hours):
10. Infants and children: low systolic blood pressure (age-specific) or less than 30% reduction in systolic blood pressure;
11. Adults: Systolic blood pressure less than 90 mm Hg or more than 30% lower than the baseline.

**7.2 Dose Delay Criteria**

In this study, no dose adjustment is required for anti-PD-1 monoclonal antibody. If a subject experiences any adverse event that requires dose interruption, the subject may suspend study treatment. The administration of Toripalimab can be postponed for up to 7 days for an adverse event and the subsequent dosing schedule will be postponed. Any dose delay of more than 7 days will be considered as a missing dose, and the subject should receive the next dose according to the pre-defined dosing schedule from the day of the first dose. If a subject has discontinued treatment for more than 56 days and the risks of continuing treatment with Toripalimab outweigh the benefits as judged by the Investigator, permanent withdrawal from study treatment should be considered. If, in the Investigator's judgment, the subject is likely to continue to gain clinical benefit from Toripalimab after discontinuation, the study drug may be resumed with the Investigator's consent. Tumor assessments for all patients were continued as per protocol even if dosing was delayed. Toripalimab administration was delayed for the following: Any grade ≥2 non-skin, treatment-related AE, with the exceptions of Grade 2 treatment-related fatigue or laboratory abnormalities do not require a treatment delay. Any AE, laboratory abnormality, or intercurrent illness which, in the judgment of the Investigator, warrants delaying the dose of study medication.

**References:**

1. Chen L: **Co-inhibitory molecules of the B7-CD28 family in the control of T-cell immunity**. *Nat Rev Immunol* 2004, **4**(5):336-347.

2. Wang FH, Ren C, Zhang Y, Yao S, Feng H, Wu H, Song HF, Zhang R, Wei XL, Xia XJ *et al*: **1186PPhase Ia study of a humanized anti-PD-1 monoclonal antibody (JS001) in Chinese patients with refractory solid tumors**. *Ann Oncol* 2017, **28**(suppl_5):mdx376.051-mdx376.051.

3. Le DT, Uram JN, Wang H, Bartlett BR, Kemberling H, Eyring AD, Skora AD, Luber BS, Azad NS, Laheru D *et al*: **PD-1 Blockade in Tumors with Mismatch-Repair Deficiency**. *N Engl J Med* 2015, **372**(26):2509-2520.

4. Le DT, Durham JN: **Mismatch repair deficiency predicts response of solid tumors to PD-1 blockade**. *Science* 2017, **357**(6349):409-413.

5. Rizvi NA, Hellmann MD, Snyder A, Kvistborg P, Makarov V, Havel JJ, Lee W, Yuan J, Wong P, Ho TS *et al*: **Cancer immunology. Mutational landscape determines sensitivity to PD-1 blockade in non-small cell lung cancer**. *Science* 2015, **348**(6230):124-128.

6. Snyder A, Makarov V, Merghoub T, Yuan J, Zaretsky JM, Desrichard A, Walsh LA, Postow MA, Wong P, Ho TS *et al*: **Genetic basis for clinical response to CTLA-4 blockade in melanoma**. *The New England journal of medicine* 2014, **371**(23):2189-2199.

7. Rosenberg JE, Hoffman-Censits J, Powles T, van der Heijden MS, Balar AV, Necchi A, Dawson N, O'Donnell PH, Balmanoukian A, Loriot Y *et al*: **Atezolizumab in patients with locally advanced and metastatic urothelial carcinoma who have progressed following treatment with platinum-based chemotherapy: a single-arm, multicentre, phase 2 trial**. *Lancet* 2016, **387**(10031):1909-1920.

8. Yarchoan M, Hopkins A, Jaffee EM: **Tumor Mutational Burden and Response Rate to PD-1 Inhibition**. *N Engl J Med* 2017, **377**(25):2500-2501.

9. Johanns TM, Miller CA, Dorward IG, Tsien C, Chang E, Perry A, Uppaluri R, Ferguson C, Schmidt RE, Dahiya S *et al*: **Immunogenomics of Hypermutated Glioblastoma: A Patient with Germline POLE Deficiency Treated with Checkpoint Blockade Immunotherapy**. *Cancer Discov*2016, **6**(11):1230-1236.

10. Mehnert JM, Panda A, Zhong H, Hirshfield K, Damare S, Lane K, Sokol L, Stein MN, Rodriguez-Rodriquez L, Kaufman HL *et al*: **Immune activation and response to pembrolizumab in POLE-mutant endometrial cancer**.*J Clin Invest* 2016, **126**(6):2334-2340.
