# Supplementary material for: A phase II clinical trial of toripalimab in advanced solid tumors with polymerase epsilon/polymerase delta (POLE/POLD1) mutation
Source: Signal Transduct Target Ther. 2024 Sep 2;9:227. doi: 10.1038/s41392-024-01939-5 (PMC11366758; doi:10.1038/s41392-024-01939-5)
Supplement: Supplementary file 2 — Supplementary file [file 41392_2024_1939_MOESM2_ESM.docx]

Supplementary Materials for

A Phase II Clinical Trial of Toripalimab in Advanced Solid Tumors with Polymerase epsilon/polymerase delta (*POLE/POLD1*) Mutation

Ying Jin, Run-Jie Huang, Wen-Long Guan, Zhi-Qiang Wang, Zong-Jiong Mai, Yu-Hong Li, Jian Xiao, Xing Zhang, Qi Zhao, Shi-Fu Chen, Ming Liu, Yan-Xia Shi, Feng Wang, Rui-Hua Xu

Correspondence: [xurh@sysucc.org.cn](mailto:xurh@sysucc.org.cn), [wangfeng@sysucc.org.cn](mailto:wangfeng@sysucc.org.cn), and [shiyx@sysucc.org.cn](mailto:shiyx@sysucc.org.cn)

**This PDF file includes:**

Figures. S1 to S5

Tables S1 to S4

**
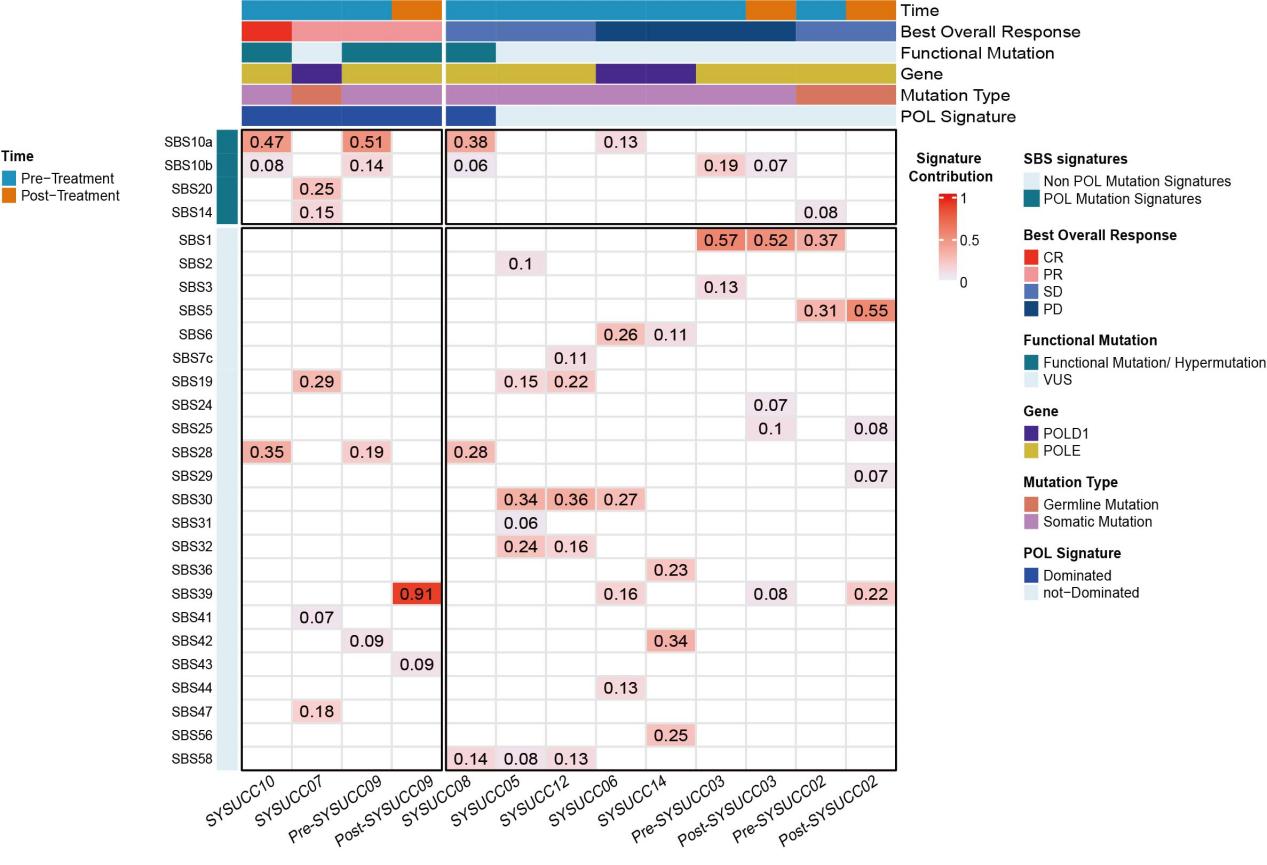
**

**Supplementary Figure 1. Tissue gene mutation signature analysis**

Heatmap of the relative attribution of *SBS* signatures. Columns represent tissue samples, and rows represent signatures. The relative attribution of signatures is displayed as black text and colors ranging from red to white in each cell.


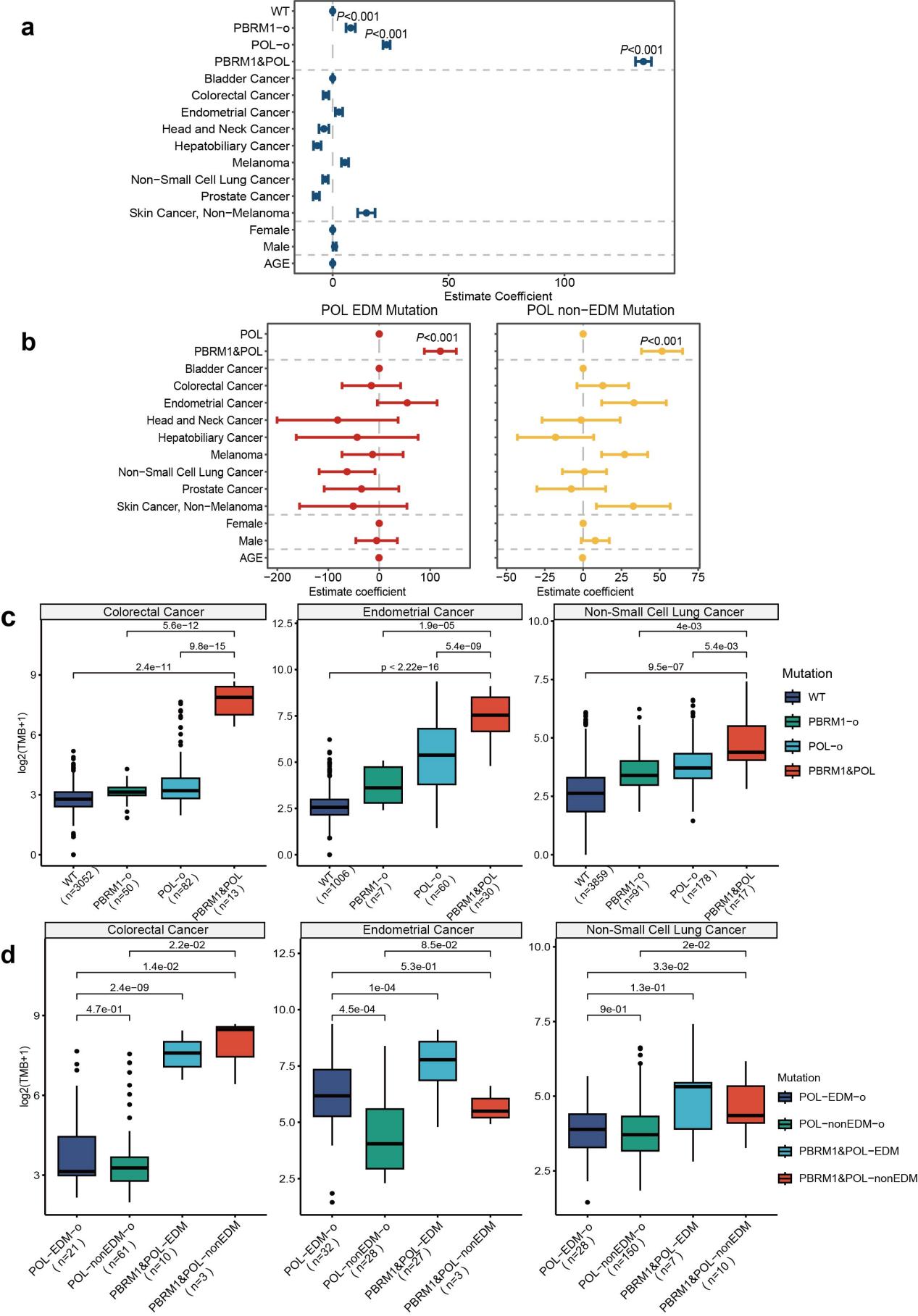


**Supplementary Figure 2. MSKCC pan-cancer cohort mutation analysis**

(a) Linear regression to compare TMB values of four groups (WT, *PBRM1*-o, *POL*-o, *PBRM1*&*POL*). (b) Linear regression to compare TMB values of the *PBRM*&*POL* and *POL*-o group stratified by mutation location. (c) TMB values for four groups (WT, *PBRM1*-o, *POL*-o, *PBRM1*&*POL*) in colorectal cancer, endometrial cancer and non-small cell lung cancer. (*P* values were calculated by wilcoxon rank sum test)

(d) TMB values for four groups (*POL*-EDM-o, *POL*-non-EDM-o, *PBRM1*&*POL*-EDM, *PBRM1*&*POL*-non-EDM ) in colorectal cancer, endometrial cancer and non-small cell lung cancer. (*P* values were calculated by wilcoxon rank sum test)


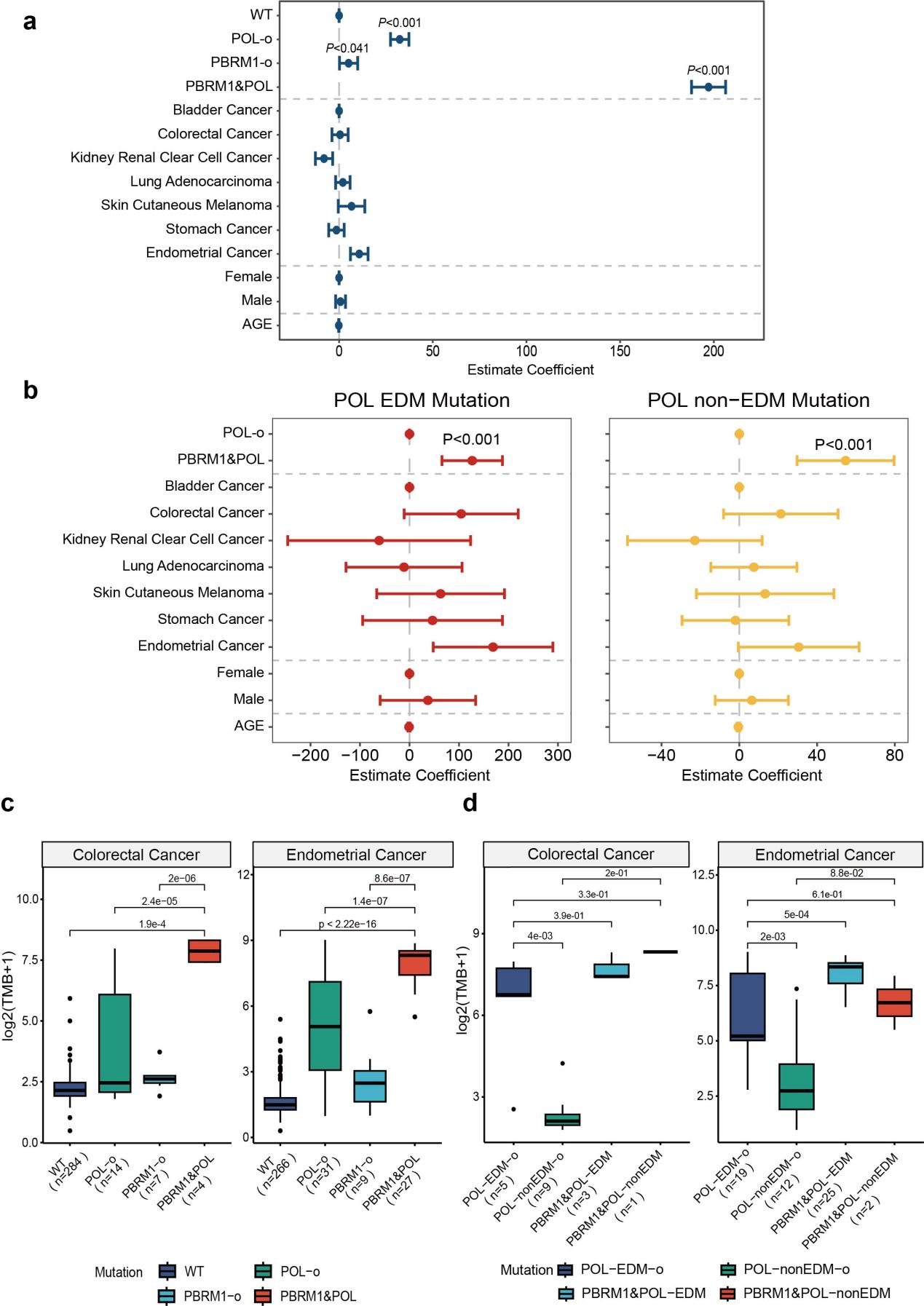


**Supplementary Figure 3. TCGA pan-cancer cohort mutation analysis**

(a) Linear regression to compare the TMB value of four groups (WT, *PBRM1*-o, *POL*-o, *PBRM1*&*POL*). (b) Linear regression to compare TMB values of the *PBRM*&*POL* and *POL*-o group stratified by mutation location. (c) TMB values for four groups (WT, *PBRM1*-o, *POL*-o, *PBRM1*&*POL*) in colorectal cancer and endometrial cancer. (*P* values were calculated by wilcoxon rank sum test) (d) TMB values for four groups (*POL*-EDM-o, *POL*-non-EDM-o, *PBRM1*&*POL*-EDM, *PBRM1*&*POL*-non-EDM ) in colorectal cancer and endometrial cancer. (*P* values were calculated by wilcoxon rank sum test)


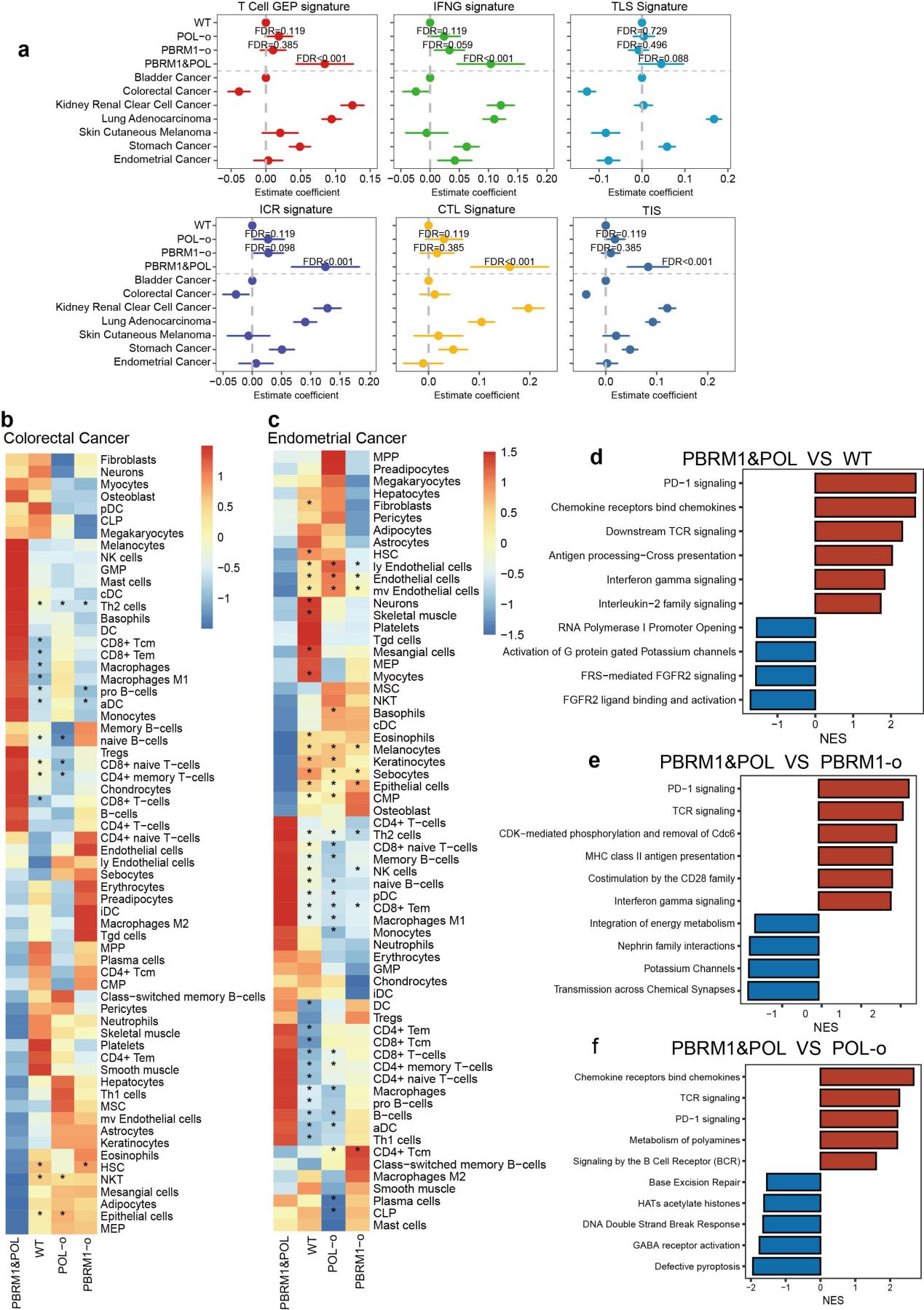


**Supplementary Figure 4. TCGA pan-cancer cohort transcriptome analysis**

(a) Linear regression to compare immunotherapy response signature score values of four groups (WT, *PBRM1*-o, *POL*-o, *PBRM1*&*POL*). (T Cell GEP: T cell-inflamed gene expression profiles; IFNG: interferon gamma; TLS: Tertiary Lymphoid Structure; ICR: Immunologic Constant of Rejection; CTL: Cytotoxic T lymphocytes; TIS: Tumor Inflammation Signature) (b-c) Heatmap of immune cell enrichment estimated by xCell in colorectal cancer and endometrial cancer. Wilcoxon rank sum test was conducted to compare the *PBRM1*&*POL* group with the other three groups (*POL*-o, *PBRM1*-o, and WT), respectively. The asterisk represents the statistical difference (*P*<0.05) between the *PBRM1*&*POL* group and the other three groups. (d-f) GSEA results for the comparison between *PBRM1*&*POL* with the other three groups, respectively.


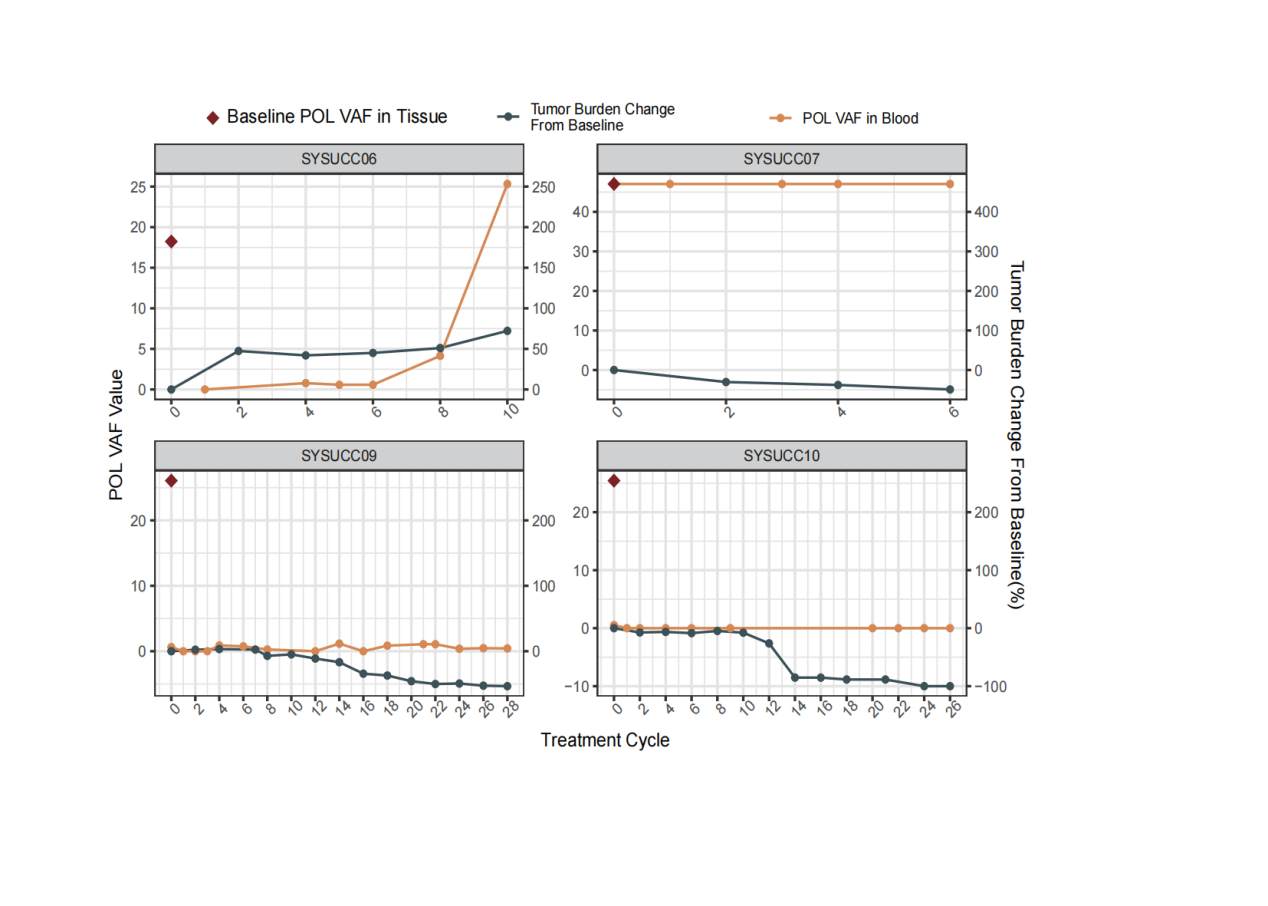


**Supplementary Figure 5. circulating DNA analysis**

Dynamic changes of *POLE/POLD1* mutations in circulating DNA of patients with more than two serum sample collections.

**Supplementary Table 1. Multivariate linear regression analysis between gene mutation and TMB in the MSKCC metastasis cancer cohort.** (Related to Supplement Figure 2a-b)

| Multivariate regression analysis between gene mutation (WT, *PBRM1*-o, *POL*-o, *PBRM1*&*POL*) and TMB adjusted cancer types and age | | | | | | |
| --- | --- | --- | --- | --- | --- | --- |
|  |  |  |  |  |  |  |
|  | Estimate | CI (lower) | CI (upper) | Std. Error | t value | Pr(>\|t\|) |
| (Intercept) | 8.37 | 6.28 | 10.46 | 1.07 | 7.86 | <0.001 |
| Mutation: *PBRM1*-o | 7.78 | 5.78 | 9.78 | 1.02 | 7.63 | <0.001 |
| Mutation: *POL*-o | 23.25 | 21.73 | 24.77 | 0.78 | 29.97 | <0.001 |
| Mutation: *PBRM1*&*POL* | 134.04 | 130.62 | 137.47 | 1.75 | 76.73 | <0.001 |
| Cancer_Type: Colorectal Cancer | -2.89 | -4.16 | -1.62 | 0.65 | -4.45 | <0.001 |
| Cancer_Type: Endometrial Cancer | 2.72 | 1.14 | 4.30 | 0.81 | 3.37 | 0.00 |
| Cancer_Type: Head and Neck Cancer | -3.79 | -5.92 | -1.66 | 1.09 | -3.49 | <0.001 |
| Cancer_Type: Hepatobiliary Cancer | -6.64 | -8.27 | -5.01 | 0.83 | -7.99 | <0.001 |
| Cancer_Type: Melanoma | 5.30 | 3.76 | 6.85 | 0.79 | 6.72 | <0.001 |
| Cancer_Type: Non-Small Cell Lung Cancer | -3.13 | -4.35 | -1.91 | 0.62 | -5.03 | <0.001 |
| Cancer_Type: Prostate Cancer | -7.07 | -8.42 | -5.71 | 0.69 | -10.22 | <0.001 |
| Cancer_Type: Skin Cancer, Non-Melanoma | 14.55 | 10.80 | 18.30 | 1.91 | 7.60 | <0.001 |
| Gender: Male | 0.84 | 0.15 | 1.53 | 0.35 | 2.38 | 0.02 |
| AGE | 0.00 | -0.02 | 0.03 | 0.01 | 0.37 | 0.71 |
|  |  |  |  |  |  |  |
| Multivariate regression analysis between gene mutation (*POL*-EDM, *PBRM1*&*POL*-EDM ) and TMB adjusted cancer types and age | | | | | | |
|  |  |  |  |  |  |  |
|  | Estimate | CI (lower) | CI (upper) | Std. Error | t value | Pr(>\|t\|) |
| (Intercept) | 94.16 | -8.47 | 196.78 | 52.36 | 1.80 | 0.07 |
| Mutation: *PBRM1*&*POL*-EDM | 119.86 | 88.51 | 151.21 | 15.99 | 7.49 | <0.001 |
| Cancer_Type: Colorectal Cancer | -15.48 | -72.82 | 41.87 | 29.26 | -0.53 | 0.60 |
| Cancer_Type: Endometrial Cancer | 54.92 | -3.63 | 113.48 | 29.88 | 1.84 | 0.07 |
| Cancer_Type: Head and Neck Cancer | -81.56 | -200.30 | 37.18 | 60.58 | -1.35 | 0.18 |
| Cancer_Type: Hepatobiliary Cancer | -43.29 | -162.80 | 76.21 | 60.97 | -0.71 | 0.48 |
| Cancer_Type: Melanoma | -13.04 | -73.05 | 46.98 | 30.62 | -0.43 | 0.67 |
| Cancer_Type: Non-Small Cell Lung Cancer | -63.10 | -117.78 | -8.42 | 27.90 | -2.26 | 0.03 |
| Cancer_Type: Prostate Cancer | -34.73 | -107.91 | 38.44 | 37.34 | -0.93 | 0.35 |
| Cancer_Type: Skin Cancer, Non-Melanoma | -51.01 | -156.41 | 54.38 | 53.78 | -0.95 | 0.34 |
| Gender: Male | -5.05 | -45.73 | 35.64 | 20.76 | -0.24 | 0.81 |
| AGE | -0.45 | -1.80 | 0.90 | 0.69 | -0.65 | 0.51 |
|  |  |  |  |  |  |  |

Multivariate regression analysis between gene mutation (*POL*-non-EDM, *PBRM1*&*POL*-non-EDM) and TMB adjusted cancer types and age

|  | Estimate | CI (lower) | CI (upper) | Std. Error | t value | Pr(>\|t\|) |
| --- | --- | --- | --- | --- | --- | --- |
| (Intercept) | 35.52 | 7.55 | 63.49 | 14.27 | 2.49 | 0.01 |
| Mutation: *PBRM1*&*POL*-non-EDM | 51.46 | 38.06 | 64.87 | 6.84 | 7.52 | <0.001 |
| Cancer_Type: Colorectal Cancer | 12.84 | -3.99 | 29.67 | 8.59 | 1.49 | 0.14 |
| Cancer_Type: Endometrial Cancer | 33.18 | 12.10 | 54.27 | 10.76 | 3.08 | 0.00 |
| Cancer_Type: Head and Neck Cancer | -1.33 | -26.77 | 24.11 | 12.98 | -0.10 | 0.92 |
| Cancer_Type: Hepatobiliary Cancer | -17.98 | -42.88 | 6.92 | 12.70 | -1.42 | 0.16 |
| Cancer_Type: Melanoma | 27.10 | 12.04 | 42.16 | 7.68 | 3.53 | <0.001 |
| Cancer_Type: Non-Small Cell Lung Cancer | 0.88 | -13.53 | 15.30 | 7.36 | 0.12 | 0.90 |
| Cancer_Type: Prostate Cancer | -7.70 | -30.13 | 14.73 | 11.45 | -0.67 | 0.50 |
| Cancer_Type: Skin Cancer, Non-Melanoma | 32.70 | 8.62 | 56.77 | 12.28 | 2.66 | 0.01 |
| Gender: Male | 7.92 | -1.22 | 17.06 | 4.66 | 1.70 | 0.09 |
| AGE | -0.38 | -0.72 | -0.04 | 0.17 | -2.19 | 0.03 |

**Supplementary Table 2. Multivariate linear regression analysis between gene mutation and TMB in the TCGA pan-cancer cohort.** (Related to Supplement Figure 3a-b)

| Multivariate regression analysis between gene mutation (WT, *PBRM1*-o, *POL*-o, *PBRM1*&*POL*) and  TMB adjusted cancer types and age | | | | | | |
| --- | --- | --- | --- | --- | --- | --- |
|  |  |  |  |  |  |  |
|  | Estimate | CI (lower) | CI (upper) | Std. Error | t value | Pr(>\|t\|) |
| (Intercept) | 14.35 | 6.39 | 22.31 | 4.06 | 3.53 | <0.001 |
| Mutation: *POL*-o | 32.36 | 27.45 | 37.27 | 2.50 | 12.92 | <0.001 |
| Mutation: *PBRM1*-o | 5.06 | 0.20 | 9.91 | 2.48 | 2.04 | 0.04 |
| Mutation: *PBRM1*&*POL* | 197.22 | 188.10 | 206.34 | 4.65 | 42.39 | <0.001 |
| Cancer_Type: COADREAD | 0.53 | -3.80 | 4.86 | 2.21 | 0.24 | 0.81 |
| Cancer_Type: KIRC | -8.04 | -12.65 | -3.43 | 2.35 | -3.42 | 0.00 |
| Cancer_Type: LUAD | 2.00 | -1.87 | 5.88 | 1.98 | 1.01 | 0.31 |
| Cancer_Type: SKCM | 6.65 | -0.42 | 13.73 | 3.61 | 1.84 | 0.07 |
| Cancer_Type: STAD | -1.41 | -5.52 | 2.70 | 2.10 | -0.67 | 0.50 |
| Cancer_Type: UCEC | 10.76 | 6.08 | 15.44 | 2.39 | 4.51 | <0.001 |
| Gender: MALE | 0.77 | -1.88 | 3.42 | 1.35 | 0.57 | 0.57 |
| AGE | -0.16 | -0.26 | -0.05 | 0.05 | -2.93 | 0.00 |
| Multivariate regression analysis between gene mutation (*POL*-EDM, *PBRM1*&*POL*-EDM) and  TMB adjusted cancer types and age | | | | | | |
|  |  |  |  |  |  |  |
|  | Estimate | CI (lower) | CI (upper) | Std. Error | t value | Pr(>\|t\|) |
| (Intercept) | 38.29 | -141.49 | 218.07 | 91.73 | 0.42 | 0.68 |
| Mutation: *PBRM1*&*POL*-EDM | 126.71 | 65.61 | 187.81 | 31.17 | 4.06 | <0.001 |
| Cancer_Type: COADREAD | 104.48 | -10.78 | 219.73 | 58.80 | 1.78 | 0.08 |
| Cancer_Type: KIRC | -61.28 | -246.07 | 123.51 | 94.28 | -0.65 | 0.52 |
| Cancer_Type: LUAD | -11.04 | -128.35 | 106.26 | 59.85 | -0.18 | 0.85 |
| Cancer_Type: SKCM | 62.89 | -66.14 | 191.91 | 65.83 | 0.96 | 0.34 |
| Cancer_Type: STAD | 46.54 | -94.77 | 187.84 | 72.10 | 0.65 | 0.52 |
| Cancer_Type: UCEC | 168.86 | 47.94 | 289.78 | 61.70 | 2.74 | 0.01 |
| Gender: MALE | 37.22 | -59.21 | 133.65 | 49.20 | 0.76 | 0.45 |
| AGE | -0.92 | -3.05 | 1.22 | 1.09 | -0.84 | 0.40 |
|  |  |  |  |  |  |  |
| Multivariate regression analysis between gene mutation (*POL*-non-EDM, *PBRM1*&*POL*-non-EDM) and  TMB adjusted cancer types and age | | | | | | |
|  |  |  |  |  |  |  |
|  | Estimate | CI (lower) | CI (upper) | Std. Error | t value | Pr(>\|t\|) |
| (Intercept) | 38.51 | -13.74 | 90.76 | 26.66 | 1.44 | 0.15 |
| Mutation: *PBRM1*&*POL*-non-EDM | 54.68 | 29.71 | 79.64 | 12.74 | 4.29 | <0.001 |
| Cancer_Type: COADREAD | 21.33 | -8.14 | 50.80 | 15.04 | 1.42 | 0.16 |
| Cancer_Type: KIRC | -22.86 | -57.49 | 11.77 | 17.67 | -1.29 | 0.20 |
| Cancer_Type: LUAD | 7.51 | -14.63 | 29.64 | 11.30 | 0.66 | 0.51 |
| Cancer_Type: SKCM | 13.27 | -22.08 | 48.63 | 18.04 | 0.74 | 0.46 |
| Cancer_Type: STAD | -2.00 | -29.45 | 25.46 | 14.01 | -0.14 | 0.89 |
| Cancer_Type: UCEC | 30.54 | -0.55 | 61.63 | 15.86 | 1.93 | 0.06 |
| Gender: MALE | 6.43 | -12.38 | 25.25 | 9.60 | 0.67 | 0.50 |
| AGE | -0.50 | -1.17 | 0.17 | 0.34 | -1.47 | 0.14 |

**Supplementary Table 3. Multivariate linear regression analysis between gene mutation and immunotherapy response signature scores in the TCGA pan-cancer cohort.** (Related to Supplement Figure 4a)

| Multivariate regression analysis between gene mutation (WT, *PBRM1*-o, *POL*-o, *PBRM1*&*POL*) and  T_Cell_GEP signature scores   \|  \| Estimate \| CI (lower) \| CI (upper) \| Std. Error \| t value \| FDR \| \| --- \| --- \| --- \| --- \| --- \| --- \| --- \| \| (Intercept) \| 0.05 \| 0.04 \| 0.06 \| 0.01 \| 9.15 \| <0.001 \| \| Mutation: *POL*-o \| 0.02 \| 0.00 \| 0.04 \| 0.01 \| 1.87 \| 0.12 \| \| Mutation: *PBRM1*-o \| 0.01 \| -0.01 \| 0.03 \| 0.01 \| 1.12 \| 0.39 \| \| Mutation: *PBRM1*&*POL* \| 0.08 \| 0.04 \| 0.13 \| 0.02 \| 4.05 \| <0.001 \| \| Cancer_Type: COADREAD \| -0.04 \| -0.05 \| -0.02 \| 0.01 \| -4.86 \| <0.001 \| \| Cancer_Type: KIRC \| 0.12 \| 0.11 \| 0.14 \| 0.01 \| 14.95 \| <0.001 \| \| Cancer_Type: LUAD \| 0.09 \| 0.08 \| 0.11 \| 0.01 \| 13.63 \| <0.001 \| \| Cancer_Type: SKCM \| 0.02 \| 0.00 \| 0.05 \| 0.01 \| 1.58 \| 0.23 \| \| Cancer_Type: STAD \| 0.05 \| 0.03 \| 0.06 \| 0.01 \| 6.42 \| <0.001 \| \| Cancer_Type: UCEC \| 0.00 \| -0.02 \| 0.02 \| 0.01 \| 0.32 \| 0.80 \|   Multivariate regression analysis between gene mutation (WT, *PBRM1*-o, *POL*-o, *PBRM1*&*POL*)  and IFN signature scores   \|  \| Estimate \| CI (lower) \| CI (upper) \| Std. Error \| t value \| FDR \| \| --- \| --- \| --- \| --- \| --- \| --- \| --- \| \| (Intercept) \| 0.16 \| 0.15 \| 0.18 \| 0.01 \| 22.21 \| <0.001 \| \| Mutation: *POL*-o \| 0.02 \| 0.00 \| 0.05 \| 0.01 \| 1.73 \| 0.12 \| \| Mutation: *PBRM1*-o \| 0.03 \| 0.01 \| 0.06 \| 0.01 \| 2.59 \| 0.06 \| \| Mutation: *PBRM1*&*POL* \| 0.10 \| 0.05 \| 0.16 \| 0.03 \| 3.55 \| <0.001 \| \| Cancer_Type: COADREAD \| -0.02 \| -0.05 \| 0.00 \| 0.01 \| -2.18 \| 0.04 \| \| Cancer_Type: KIRC \| 0.12 \| 0.10 \| 0.14 \| 0.01 \| 10.39 \| <0.001 \| \| Cancer_Type: LUAD \| 0.11 \| 0.09 \| 0.13 \| 0.01 \| 11.31 \| <0.001 \| \| Cancer_Type: SKCM \| -0.01 \| -0.04 \| 0.03 \| 0.02 \| -0.32 \| 0.75 \| \| Cancer_Type: STAD \| 0.06 \| 0.04 \| 0.08 \| 0.01 \| 5.88 \| <0.001 \| \| Cancer_Type: UCEC \| 0.04 \| 0.01 \| 0.07 \| 0.01 \| 2.89 \| 0.01 \|   Multivariate regression analysis between gene mutation (WT, *PBRM1*-o, *POL*-o, *PBRM1*&*POL*) and  TLS signature scores   \|  \| Estimate \| CI (lower) \| CI (upper) \| Std. Error \| t value \| FDR \| \| --- \| --- \| --- \| --- \| --- \| --- \| --- \| \| (Intercept) \| -0.05 \| -0.06 \| -0.04 \| 0.01 \| -7.47 \| <0.001 \| \| Mutation: *POL*-o \| 0.00 \| -0.02 \| 0.03 \| 0.01 \| 0.35 \| 0.73 \| \| Mutation: *PBRM1*-o \| -0.01 \| -0.03 \| 0.01 \| 0.01 \| -0.68 \| 0.50 \| \| Mutation: *PBRM1*&*POL* \| 0.04 \| -0.01 \| 0.10 \| 0.03 \| 1.71 \| 0.09 \| \| Cancer_Type: COADREAD \| -0.13 \| -0.15 \| -0.11 \| 0.01 \| -12.75 \| <0.001 \| \| Cancer_Type: KIRC \| 0.00 \| -0.02 \| 0.02 \| 0.01 \| 0.33 \| 0.74 \| \| Cancer_Type: LUAD \| 0.17 \| 0.15 \| 0.18 \| 0.01 \| 19.17 \| <0.001 \| \| Cancer_Type: SKCM \| -0.08 \| -0.12 \| -0.05 \| 0.02 \| -5.12 \| <0.001 \| \| Cancer_Type: STAD \| 0.06 \| 0.04 \| 0.08 \| 0.01 \| 6.07 \| <0.001 \| \| Cancer_Type: UCEC \| -0.08 \| -0.10 \| -0.05 \| 0.01 \| -5.89 \| <0.001 \|   Multivariate regression analysis between gene mutation (WT, *PBRM1*-o, *POL*-o, *PBRM1*&*POL*) and  ICR signature scores   \|  \| Estimate \| CI (lower) \| CI (upper) \| Std. Error \| t value \| FDR \| \| --- \| --- \| --- \| --- \| --- \| --- \| --- \| \| (Intercept) \| -0.10 \| -0.12 \| -0.09 \| 0.01 \| -13.94 \| <0.001 \| \| Mutation: *POL*-o \| 0.03 \| 0.00 \| 0.05 \| 0.01 \| 1.94 \| 0.12 \| \| Mutation: *PBRM1*-o \| 0.03 \| 0.00 \| 0.05 \| 0.01 \| 2.14 \| 0.10 \| \| Mutation: *PBRM1*&*POL* \| 0.13 \| 0.07 \| 0.18 \| 0.03 \| 4.25 \| <0.001 \| \| Cancer_Type: COADREAD \| -0.03 \| -0.05 \| -0.01 \| 0.01 \| -2.49 \| 0.02 \| \| Cancer_Type: KIRC \| 0.13 \| 0.11 \| 0.15 \| 0.01 \| 10.98 \| <0.001 \| \| Cancer_Type: LUAD \| 0.09 \| 0.07 \| 0.11 \| 0.01 \| 9.27 \| <0.001 \| \| Cancer_Type: SKCM \| -0.01 \| -0.04 \| 0.03 \| 0.02 \| -0.34 \| 0.75 \| \| Cancer_Type: STAD \| 0.05 \| 0.03 \| 0.07 \| 0.01 \| 4.73 \| <0.001 \| \| Cancer_Type: UCEC \| 0.01 \| -0.02 \| 0.04 \| 0.01 \| 0.44 \| 0.80 \|   Multivariate regression analysis between gene mutation (WT, *PBRM1*-o, *POL*-o, *PBRM1*&*POL*) and  CTL signature scores   \|  \| Estimate \| CI (lower) \| CI (upper) \| Std. Error \| t value \| FDR \| \| --- \| --- \| --- \| --- \| --- \| --- \| --- \| \| (Intercept) \| -0.23 \| -0.25 \| -0.21 \| 0.01 \| -23.52 \| <0.001 \| \| Mutation: *POL*-o \| 0.03 \| -0.01 \| 0.07 \| 0.02 \| 1.65 \| 0.12 \| \| Mutation: *PBRM1*-o \| 0.02 \| -0.02 \| 0.05 \| 0.02 \| 0.99 \| 0.39 \| \| Mutation: *PBRM1*&*POL* \| 0.16 \| 0.08 \| 0.24 \| 0.04 \| 4.12 \| <0.001 \| \| Cancer_Type: COADREAD \| 0.01 \| -0.02 \| 0.04 \| 0.01 \| 0.81 \| 0.42 \| \| Cancer_Type: KIRC \| 0.20 \| 0.17 \| 0.23 \| 0.02 \| 12.74 \| <0.001 \| \| Cancer_Type: LUAD \| 0.10 \| 0.08 \| 0.13 \| 0.01 \| 8.11 \| <0.001 \| \| Cancer_Type: SKCM \| 0.02 \| -0.03 \| 0.07 \| 0.02 \| 0.80 \| 0.63 \| \| Cancer_Type: STAD \| 0.05 \| 0.02 \| 0.08 \| 0.01 \| 3.44 \| <0.001 \| \| Cancer_Type: UCEC \| -0.01 \| -0.05 \| 0.03 \| 0.02 \| -0.55 \| 0.80 \|   Multivariate regression analysis between gene mutation (WT, *PBRM1-*o*, POL-*o*,PBRM1*&*POL*) and  TIS signature scores   \|  \| Estimate \| CI (lower) \| CI (upper) \| Std. Error \| t value \| FDR \| \| --- \| --- \| --- \| --- \| --- \| --- \| --- \| \| (Intercept) \| 0.03 \| 0.02 \| 0.04 \| 0.01 \| 6.11 \| <0.001 \| \| Mutation: *POL*-o \| 0.02 \| 0.00 \| 0.04 \| 0.01 \| 1.86 \| 0.12 \| \| Mutation: *PBRM1*-o \| 0.01 \| -0.01 \| 0.03 \| 0.01 \| 1.09 \| 0.39 \| \| Mutation: *PBRM1&POL* \| 0.08 \| 0.04 \| 0.12 \| 0.02 \| 4.04 \| <0.001 \| \| Cancer_Type: COADREAD \| -0.04 \| -0.05 \| -0.02 \| 0.01 \| -4.88 \| <0.001 \| \| Cancer_Type: KIRC \| 0.12 \| 0.10 \| 0.14 \| 0.01 \| 14.70 \| <0.001 \| \| Cancer_Type: LUAD \| 0.09 \| 0.08 \| 0.11 \| 0.01 \| 13.49 \| <0.001 \| \| Cancer_Type: SKCM \| 0.02 \| 0.00 \| 0.05 \| 0.01 \| 1.58 \| 0.23 \| \| Cancer_Type: STAD \| 0.05 \| 0.03 \| 0.06 \| 0.01 \| 6.34 \| <0.001 \| \| Cancer_Type: UCEC \| 0.00 \| -0.02 \| 0.02 \| 0.01 \| 0.26 \| 0.80 \|   **Supplementary Table 4. The values of *POL* VAF of cancer tissue in baseline, *POL* VAF in blood and tumor burden change from baseline for each patient**. (Related to Supplement Figure 5) |
| --- | --- | --- | --- | --- | --- | --- | --- | --- | --- | --- | --- | --- | --- | --- | --- | --- | --- | --- | --- | --- | --- | --- | --- | --- | --- | --- | --- | --- | --- | --- | --- | --- | --- | --- | --- | --- | --- | --- | --- | --- | --- | --- | --- | --- | --- | --- | --- | --- | --- | --- | --- | --- | --- | --- | --- | --- | --- | --- | --- | --- | --- | --- | --- | --- | --- | --- | --- | --- | --- | --- | --- | --- | --- | --- | --- | --- | --- | --- | --- | --- | --- | --- | --- | --- | --- | --- | --- | --- | --- | --- | --- | --- | --- | --- | --- | --- | --- | --- | --- | --- | --- | --- | --- | --- | --- | --- | --- | --- | --- | --- | --- | --- | --- | --- | --- | --- | --- | --- | --- | --- | --- | --- | --- | --- | --- | --- | --- | --- | --- | --- | --- | --- | --- | --- | --- | --- | --- | --- | --- | --- | --- | --- | --- | --- | --- | --- | --- | --- | --- | --- | --- | --- | --- | --- | --- | --- | --- | --- | --- | --- | --- | --- | --- | --- | --- | --- | --- | --- | --- | --- | --- | --- | --- | --- | --- | --- | --- | --- | --- | --- | --- | --- | --- | --- | --- | --- | --- | --- | --- | --- | --- | --- | --- | --- | --- | --- | --- | --- | --- | --- | --- | --- | --- | --- | --- | --- | --- | --- | --- | --- | --- | --- | --- | --- | --- | --- | --- | --- | --- | --- | --- | --- | --- | --- | --- | --- | --- | --- | --- | --- | --- | --- | --- | --- | --- | --- | --- | --- | --- | --- | --- | --- | --- | --- | --- | --- | --- | --- | --- | --- | --- | --- | --- | --- | --- | --- | --- | --- | --- | --- | --- | --- | --- | --- | --- | --- | --- | --- | --- | --- | --- | --- | --- | --- | --- | --- | --- | --- | --- | --- | --- | --- | --- | --- | --- | --- | --- | --- | --- | --- | --- | --- | --- | --- | --- | --- | --- | --- | --- | --- | --- | --- | --- | --- | --- | --- | --- | --- | --- | --- | --- | --- | --- | --- | --- | --- | --- | --- | --- | --- | --- | --- | --- | --- | --- | --- | --- | --- | --- | --- | --- | --- | --- | --- | --- | --- | --- | --- | --- | --- | --- | --- | --- | --- | --- | --- | --- | --- | --- | --- | --- | --- | --- | --- | --- | --- | --- | --- | --- | --- | --- | --- | --- | --- | --- | --- | --- | --- | --- | --- | --- | --- | --- | --- | --- | --- | --- | --- | --- | --- | --- | --- | --- | --- | --- | --- | --- | --- | --- | --- | --- | --- | --- | --- | --- | --- | --- | --- | --- | --- | --- | --- | --- | --- | --- | --- | --- | --- | --- | --- | --- | --- | --- | --- | --- | --- | --- | --- | --- | --- | --- | --- | --- | --- | --- | --- | --- | --- | --- | --- | --- | --- | --- | --- | --- | --- | --- | --- | --- | --- | --- | --- | --- | --- | --- | --- | --- | --- | --- | --- | --- | --- | --- | --- | --- | --- | --- | --- | --- | --- | --- | --- |
|  |

| ID | Treatment Cycle | *POL* VAF in Blood (%) | Tumor Burden Change From Baseline (%) | Baseline *POL* VAF in Tissue (%) |
| --- | --- | --- | --- | --- |
| SYSUCC06 | 0 | NA | 0 | 18.23 |
| SYSUCC06 | 1 | 0 | NA |  |
| SYSUCC06 | 2 | NA | 47.43202 |  |
| SYSUCC06 | 4 | 0.78 | 41.99396 |  |
| SYSUCC06 | 5 | 0.58 | NA |  |
| SYSUCC06 | 6 | 0.58 | 45.01511 |  |
| SYSUCC06 | 8 | 4.14 | 51.0574 |  |
| SYSUCC06 | 10 | 25.32 | 72.20544 |  |
| SYSUCC07 | 0 | 47.05 | 0 | 47.05 |
| SYSUCC07 | 1 | 47.05 | NA |  |
| SYSUCC07 | 2 | NA | -30.4 |  |
| SYSUCC07 | 3 | 47.05 | NA |  |
| SYSUCC07 | 4 | 47.05 | -37.84 |  |
| SYSUCC07 | 6 | 47.05 | -49.2 |  |
| SYSUCC09 | 0 | 0.63 | 0 | 26.07 |
| SYSUCC09 | 1 | 0 | NA |  |
| SYSUCC09 | 2 | 0 | 2.290076 |  |
| SYSUCC09 | 3 | 0 | NA |  |
| SYSUCC09 | 4 | 0.89 | 3.307888 |  |
| SYSUCC09 | 6 | 0.75 | NA |  |
| SYSUCC09 | 7 | NA | 2.544529 |  |
| SYSUCC09 | 8 | 0.27 | -7.12468 |  |
| SYSUCC09 | 10 | NA | -4.83461 |  |
| SYSUCC09 | 12 | 0 | -11.1959 |  |
| SYSUCC09 | 14 | 1.16 | -16.7939 |  |
| SYSUCC09 | 16 | 0 | -34.3511 |  |
| SYSUCC09 | 18 | 0.85 | -37.1501 |  |
| SYSUCC09 | 20 | NA | -45.8015 |  |
| SYSUCC09 | 21 | 1.07 | NA |  |
| SYSUCC09 | 22 | 1.07 | -50.1272 |  |
| SYSUCC09 | 24 | 0.38 | -49.3639 |  |
| SYSUCC09 | 26 | 0.46 | -52.6718 |  |
| SYSUCC09 | 28 | 0.43 | -53.4351 |  |
| SYSUCC09 | 29 | NA | -64.631 |  |
| SYSUCC09 | 31 | NA | -73.028 |  |
| SYSUCC10 | 0 | 0.52 | 0 | 25.43 |
| SYSUCC10 | 1 | 0 | NA |  |
| SYSUCC10 | 2 | 0 | -7.38916 |  |
| SYSUCC10 | 4 | 0 | -6.40394 |  |
| SYSUCC10 | 6 | 0 | -8.53859 |  |
| SYSUCC10 | 8 | NA | -4.7619 |  |
| SYSUCC10 | 9 | 0 | NA |  |
| SYSUCC10 | 10 | NA | -7.71757 |  |
| SYSUCC10 | 12 | NA | -26.2726 |  |
| SYSUCC10 | 14 | NA | -85.2217 |  |
| SYSUCC10 | 16 | NA | -85.2217 |  |
| SYSUCC10 | 18 | NA | -88.5057 |  |
| SYSUCC10 | 20 | 0 | NA |  |
| SYSUCC10 | 21 | NA | -88.5057 |  |
| SYSUCC10 | 22 | 0 | NA |  |
| SYSUCC10 | 24 | 0 | -100 |  |
| SYSUCC10 | 26 | 0 | -100 |  |

* NA in *POL* VAF in Blood (%) indicates no qualified ctDNA data at this treatment cycles, and NA in Tumor Burden Change From Baseline (%) indicates no efficacy evaluation at this treatment cycle.
