# Supplementary material for: A phase II clinical trial of toripalimab in advanced solid tumors with polymerase epsilon/polymerase delta (POLE/POLD1) mutation
Source: Signal Transduct Target Ther. 2024 Sep 2;9:227. doi: 10.1038/s41392-024-01939-5 (PMC11366758; doi:10.1038/s41392-024-01939-5)
Supplement: Supplementary file 3 — Informed Consent Form [file 41392_2024_1939_MOESM3_ESM.doc]

| Research center number____ | Patient initials____ | Random number____ |
| --- | --- | --- |

**Informed Consent Form**

**Background**

Immunotherapy has made significant progress as a novel treatment for malignant tumours in recent years. In 2018, the Nobel Prize in Physiology or Medicine was awarded to American immunologist James P Alison and Japanese immunologist Tasuku Honjo for their contributions to the field of tumor immunity. The Food and Drug Administration (FDA) first approved cell immunotherapy for prostate cancer in 2010 and then approved the first immune checkpoint antagonist to be marketed worldwide in 2011- the monoclonal antibody Ipilimumab (trade name: Yervoy) from Squibb targeting CTLA-4 (cytotoxic T lymphocyte-associated antigen-4). In 2014, Nivolumab (trade name: Opdivo), a PD-1 (programmed cell death protein 1) inhibitor jointly developed by Ono Pharmaceutical and Squibb, and Pembrolizumab (trade name: Keytruda), a PD-1 inhibitor developed by Merck&Co., Ltd., were also approved for marketing. At present, immunotherapy for tumors has become one of the clinical treatment options, mainly including cytokine therapy, therapeutic vaccines, adoptive T-cell therapy (ACT), and immune checkpoint blockade or immune co-stimulatory pathway activators including CTLA-4/PD-1/PD-L1 (programmed cell death ligand 1) antibody drugs. In recent years, numerous clinical studies have shown that checkpoint-specific targeted monoclonal antibody drugs have shown good efficacy when used alone or in combination with chemotherapy, elevating the status of immunotherapy in treating various tumors.

PD-1 is an important inhibitory receptor mainly expressed on the surface of T cells, B cells, monocytes, and NK cells. The ligands of PD-1 are B7 homologous proteins PD-L1 (also known as B7-H1) and PD-L2 (also known as B7-DC). PD-L1 is widely expressed in various types of tumor cells and immune cells. The immune receptor tyrosine-based switch motif (ITSM) in the intracellular segment of PD-1 mediates the recruitment of SHP family phosphatases and the inhibition of T cell activation signals. The binding of PD-1/PD-L1 plays a vital role in downregulating T cell activation and maintaining peripheral immune tolerance, while tumor cells can inhibit T cell activation and evade immune cell killing by expressing PD-L1 and interacting with PD-1.

At present, clinical trials of targeted drugs for PD-1 at home and abroad include advanced melanoma, non-small cell lung cancer, renal cell cancer, gastric cancer, ovarian cancer, triple-negative breast cancer, colorectal cancer, pancreatic adenocarcinoma, hepatocellular carcinoma, prostate cancer, transitional cell cancer, head and neck squamous cell carcinoma, head and neck cancer, adult acute myeloid leukaemia, chronic myeloid leukaemia, multiple myeloma, myelodysplastic syndrome, cervical cancer, and glioblastoma. The completed clinical trials and interim results of some trials indicate that anti-PD-1 antibody drugs have significant advantages over previous treatment methods in terms of efficacy and safety.

JS001 is a neutralizing antibody targeting human PD-1 developed by Shanghai Junshi Biosciences Co., Ltd. It binds to PD-1 with high affinity, selectively blocking the interaction of PD-1 with its ligands PD-L1 and PD-L2, thereby activating T lymphocytes, increasing lymphocyte proliferation, and cytokine secretion, especially IFN-γ.

Preclinical efficacy trials have confirmed that JS001 significantly stimulates the proliferation of CD4+and CD8+T cells and promotes the activation of effector/memory T cells in an animal model of graft-versus-host reaction (GVHD) induced by adoptive transfer of human PBMCs. At the same time, research on the transplantation of human melanoma 624 MEL animal model has shown that the combination of JS001 and cytotoxic lymphocytes (CTLs) can eliminate immunosuppressive effects, promote the killing effect of CTLs on tumor cells, and achieve the expected good therapeutic effect. Compared with nivolumab and pembrolizumab, the differences in CDR sequences and structures between JS001 and the antigen binding site are mainly reflected in the differences in six CDR sequences, which determine the different physicochemical and biological characteristics of the three. Based on the affinity detection results of SPR or ELISA methods, JS001 has a higher affinity than pembrolizumab and nivolumab.

JS001 is conducting three Phase Ia studies in China (CTR20160274, CTR20160187, CTR20160176). The first human study, JS001-I-CRP-1.3, conducted at the Sun Yat-sen University Cancer Center, was a dose escalation study which followed the 3+3 principle. The patients received continuous dosing treatments of 0.3mg/kg, 1mg/kg, 3mg/kg, and 10mg/kg once every two weeks. According to the current first human study data of JS001, no DLT was observed. The existing safety and pharmacokinetic data demonstrate that the safety of JS001 in patients is acceptable. The research results indicate that JS001 has shown preliminary efficacy in patients with gastric cancer, esophageal cancer, nasopharyngeal cancer, and head and neck cancer.

Mismatch repair (MMR) gene status is currently recognized as a biomarker for predicting the efficacy of anti-PD-1 therapy. Previous clinical studies have shown that solid tumors with mismatch repair deficiency (dMMR) have an objective response rate of 53% when treated with anti-PD-1 therapy. In addition, the predictive role of tumor mutation burden in the efficacy of anti-PD-1 therapy is increasingly emphasized. In 2015, a study showed that PD-1/PD-L1 antibodies were significantly more effective in lung cancer patients with high tumor mutation burden than in lung cancer patients with low tumor mutation burden. In addition, a high tumor mutation burden can also help predict the efficacy of tumor immunotherapy in bladder cancer and melanoma. In 2017, a study based on tumor types showed that the higher tumor mutation burden, the higher the objective response rate of anti-PD-1 treatment. Our team’s preliminary studies have shown that mutations in the POLE gene may increase the mutation burden in the tumor with proficient mismatch repair genes (pMMR). The effectiveness of anti-PD-1 therapy in malignant gliomas and endometrial cancers with mutations in the POLE gene has been reported in case reports.

This study is a single-arm, open-label, phase II clinical trial clinical trial sponsored by the Sun Yat-sen University Cancer Center. It aims to investigate the overall efficacy (complete response [CR] and partial response [PR]) of JS001 in the treatment of advanced solid tumor patients with POLE/POLD gene mutations and high microsatellite instability, providing a basis for further large-scale clinical research.

If you agree to participate in this study, you have the right to know the specific steps you will go through. The following information is provided to help you understand the potential benefits and risks of participating in the study. After fully understanding this information, you can decide whether to participate independently. Participating in research is your voluntary act, not your obligation. You can agree to participate or not.

The Independent Ethics Committee (IEC) is the department responsible for protecting patients’ rights participating in this study at our hospital. The ethics committee of your doctor's medical center has also approved this research project.

**Research objective**

Primary objective: To investigate the overall efficacy (complete response [CR] and partial response [PR]) of JS001 in Chinese patients with advanced solid tumors and MSS or MSI-L with POLE/POLD gene mutations.

Secondary objective: To investigate the overall survival (OS), progression-free survival (PFS), and safety of JS001 in Chinese patients with advanced solid tumors and MSS or MSI-L with POLE/POLD gene mutations.

Exploratory endpoint: Collect blood, pathological slides, saliva, and fecal samples to further explore the factors affecting the efficacy of immunotherapy for MSS or MSI-L advanced solid tumors with POLE or POLD gene mutations.

**Treatment plan**

If you agree to participate in the study, you will receive the following treatment plan: JS001 treatment: intravenous infusion of JS001 240mg on the first day, repeated every three weeks.

Treatment continues until the patient develops intolerance to toxicity, disease progression, death, or refuses to continue participating in clinical research. If the above situation does not occur, it is recommended that the patient continue treatment for one year. After that, the researchers will communicate with the patient and formulate a treatment plan for the patient until the disease progresses.

If the following situations occur, the doctor will terminate your treatment with the investigational drug:

1. Any other treatments specific to your disease or concomitant treatments with other investigational drugs were used during the trial period.

2. Receive radiotherapy except for local radiotherapy taken to control bone pain or other reasons.

3. You feel that you cannot tolerate the adverse reactions of the investigational drug.

4. Disease progression, in which case the doctor will discuss further treatment with you.

5. The doctor informs you that treatment needs to be stopped.

**Research steps**

If you agree to participate in the study, the doctor will inquire about your medical history and current condition, perform blood and urine tests, electrocardiography, CT or magnetic resonance imaging, etc., according to clinical practice, and determine whether you meet the requirements of the study according to the provisions in the study protocol. Doctors will provide a standard treatment plan for patients with the advanced stage of initial diagnosis who have not received standard treatment. If you voluntarily give up standard treatment, you may also be screened for this clinical trial.

If your conditions meet the research criteria, you will receive treatment. After starting treatment, the doctor will collect information on adverse events after your medication until 30 days after the last trial administration. Hematological tests will be performed every 1-2 weeks, and CT or MRI scans every six weeks (± 2 weeks). After the treatment is completed and before the doctor notifies you of the end of the study, please come for a follow-up every two months and receive haematological, CT, or MR examinations. You need to undergo a tumor tissue biopsy or provide ten tumor tissue slides for genetic testing before treatment. Blood, stool, and saliva samples need to be collected before treatment and during each efficacy evaluation for exploratory research. The exploratory study collected specimens free of charge and at no patient cost.

**Precautions**

You should be aware of the following:

In most cases, treatment and examination will be carried out according to the abovementioned conventional regulations. But if your doctor deems it necessary, they will conduct additional tests anytime.

If you participate in the study, please follow the doctor's treatment arrangements. If you experience any discomfort or adverse reactions, please inform your doctor promptly. If the doctor decides to stop research treatment, he/she will discuss the next step of the treatment plan with you.

At the end of the research treatment, you need to undergo a physical examination, blood test, chest X-ray, magnetic resonance imaging, and ultrasound examination.

After completing the research treatment, your doctor will keep in touch with you.

If you participate in a study, be sure to tell your doctor about your other medications, whether you have seen another doctor, received new treatment, participated in other clinical studies, or whether your feelings have changed since the last follow-up.

If other doctors invite you to participate in other clinical trials, please inform them that you currently participate in this study.

**Pregnancy/contraception**

If you believe you are pregnant or may become pregnant during the research process, you should not participate in this study. Therefore, your doctor will verify that you are indeed using a reliable contraceptive method before starting medication research.

For women: women who are likely to become pregnant must undergo pregnancy tests within eight days before the start of the first cycle of treatment in the trial. If there is a suspicion of contraceptive failure or a change in the menstrual cycle, pregnancy tests must be repeated. You must immediately inform your doctor if you become pregnant during the study period.

For men: because the effects of the drugs used in this study on infants are not yet known, you and your spouse must take contraceptive measures during the trial period. If your spouse becomes pregnant during your medication period or within 90 days of discontinuing medication, you must notify your doctor.

**Risk**

Any research may have certain risks and discomforts. During the study, slight pain or bruising may occur when blood is drawn and a tumor puncture is performed. The use of peripheral intravenous infusion itself can cause temporary irritation and congestion at the injection site. Research medication may also have side effects. The following are potential side effects discovered in previous studies: common adverse reactions of JS001 include cough, nausea, decreased appetite, fatigue, rash, constipation, weight loss, anemia, fever, elevated alanine aminotransferase, elevated aspartate aminotransferase, elevated blood glucose, elevated blood bilirubin, urine leukocytes positive, urine protein positive, decreased leukocyte count, hyponatremia, hypothyroidism, etc. In addition, as the research drug is a recombinant humanized monoclonal antibody, such drugs may cause allergic reactions, infusion reactions, and immune-related adverse reactions. In previous studies, patients have experienced these side effects, but you may also experience other unpredictable side effects.

These side effects may cause minor inconvenience or be serious, but if any side effects occur, the doctor responsible for you will closely monitor you.

Although the treatment is effective for your illness, these side effects may still occur.

We will regularly evaluate the medication's effectiveness. If no efficacy is observed, treatment will be discontinued.

Your doctor may exclude you from the study if the treatment is harmful to you if you have not followed the treatment guidelines if it is found that you do not meet the trial requirements, or if the study is cancelled.

You will be notified promptly if any new information related to the study drug appears during the research process that may affect your decision to continue participating.

**Benefits**

The data obtained from your treatment process will enable doctors to treat patients like you better, which will be very helpful for future patients.

Your tumor may shrink or be under control, survival may be prolonged, and quality of life may be improved. However, like most treatments, the treatment in this study may not be able to control your disease.

If you participate in this study, you will receive free access to the research drug JS001 and free testing for the POLE/POLD gene.

**Research-related compensation and treatment**

During the research process, if abnormalities caused by the disease or non-drug factors require medical care, your doctor will examine them and provide the necessary treatment. The damages caused by your participation in this study will be carefully evaluated and treated appropriately to ensure the benefits and treatment you deserve. They will be handled and compensated following relevant Chinese regulations. However, this does not include damages caused by your failure to comply with this informed consent form, protocol, or instructions given to you by the researchers during your participation in this clinical trial. You will not lose any legal rights by signing this informed consent form.

**Confidentiality**

Your privacy will be protected. All information related to your privacy collected in this study will be kept confidential under relevant regulations. All information about you or your health output from the doctor's clinic/hospital will be numbered, and your true identity will not be disclosed. Your identity will not be recorded in any database, speech, or article.

During the study period or for up to 15 years after the end of the study, in addition to the study physician, the sponsor or its authorized representative, members of the ethics committee, and personnel from Chinese health supervision agencies (such as the China Food and Drug Administration) may have direct access to your medical records by local regulatory requirements, and they may have access to your identification information. This type of inspection ensures that the research is conducted correctly and that the data is high quality. These people who see records showing your true identity will keep it confidential.

**Participate/withdraw**

Whether or not to participate in this study is entirely up to you. Even if you refuse to participate in this study, you will not be adversely affected, including the medical treatment and care you should receive. If you decide to participate, you will receive and sign this informed consent form. If you choose to participate, you can still withdraw from the study at any time. Withdrawing from the study will not affect the treatment you should receive.

In addition, if the research physician considers continuing to participate in the study is no longer in your best interest, they may withdraw you.

If you terminate the study treatment, doctors can still obtain follow-up information from your future medical history records.

The sponsor may permanently suspend this study without seeking your prior consent. In this case, the doctor will notify you and discuss further treatment issues.

Before you sign the consent form, if there is anything in this document you do not understand or have any questions, please consult your doctor. Before deciding whether to participate in the study, please carefully read the entire document and discuss it with your doctor or anyone you think needs to be consulted, such as your family members. Only after you sign and date the consent form can your doctor conduct a comprehensive evaluation to determine whether you are suitable to participate in the study.

**Consultation on Content**

The Ethics Committee of the Sun Yat-sen University Cancer Center has reviewed this study. If you have any questions about your rights, please contact the Ethics Committee at __________, phone: 020-87343009. If you have any questions about the use of the investigational drug or related examinations, please contact your attending physician at _______________, phone number: ________________.

I now declare that I have read the patient information of the study mentioned above (research title: Phase II clinical study of JS001 treatment for advanced solid tumors with POLE gene mutations and mismatch repair proficiency)

- I have understood this study's purpose, expected benefits, and risks. I have understood that research doctors are responsible for providing me with any other information regarding the research itself and the harm it may cause.
- I have understood that I am voluntarily participating in the study, and I may refuse to participate and withdraw my consent at any time, and cease participation without any punishment or loss of any other benefits I may have.
- Within the scope of the research, I agree that the researchers and the sponsor will collect and process research data, including information on my health condition. I agree that the research data can be processed in confidentiality by the research centre staff, the sponsor's authorised personnel, and the health regulatory department personnel. I agree that the sponsor or its authorized representative may directly access and obtain my original medical records to verify the procedures and information of the clinical study, and this process will also be confidential. I agree that the collected data about me can still be used even if I withdraw from the experiment.
- I now sign this consent form to indicate my voluntary participation in this study. I have realized that the research treatment used is experimental and carries risks.
- My name or any information identifying me as a research participant will not be disclosed except as required by laws and regulations or authorized by myself/my legal representative.

I declare that I have truthfully answered the doctor's questions about my medical history and agree to accept the arrangements made by the research doctor for me. After signing the consent form, I will receive an original copy.

Patient Name (in regular script) ______ Patient Signature ______ Date__________

If the patient has designated a legal representative (if applicable):

Name of client (in regular script) _____Signature of client________ Date_________

Witness (if applicable)

Witness Name (in regular script)_______Signature of Principal ______Date________

Name of Researcher (in regular script) ___Signature of Researcher____ Date_______
